# Percutaneous Coronary Intervention for Chronic Total Occlusions Modulates Cardiac Hypoxic and Inflammatory Stress

**DOI:** 10.3390/jcm15020517

**Published:** 2026-01-08

**Authors:** Luis Carlos Maestre-Luque, Rafael Gonzalez-Manzanares, Ignacio Gallo, Francisco Hidalgo, Javier Suárez de Lezo, Miguel Romero, Simona Espejo-Perez, Carlos Perez-Sanchez, Julio Manuel Martínez-Moreno, Rafael González-Fernandez, Manuel Pan, Soledad Ojeda

**Affiliations:** 1 Department of Cardiology, Reina Sofia University Hospital, 14002 Córdoba, Spain; lmaestreluque@gmail.com (L.C.M.-L.); nachogallofernandez@gmail.com (I.G.); fjhl.87@gmail.com (F.H.); jslht@yahoo.es (J.S.d.L.); miguelromeromoreno960@gmail.com (M.R.); manuelpanalvarez@gmail.com (M.P.); soledad.ojeda18@gmai.com (S.O.); 2 Maimonides Biomedical Research Institute of Cordoba (IMIBIC), 14002 Córdoba, Spain; b32pesac@uco.es (C.P.-S.); julio@cobiomic.com (J.M.M.-M.); 3 Department of Medical and Surgical Sciences, University of Cordoba, 14002 Córdoba, Spain; 4 Centro de Investigación Biomédica en Red Enfermedades Cardiovasculares (CIBERCV), 28029 Madrid, Spain; 5 Department of Radiology, Reina Sofia University Hospital, 14002 Córdoba, Spain; simona.espejo.sspa@juntadeandalucia.es; 6 Cobiomic Bioscience SL, EBT Maimonides Biomedical Research Institute of Cordoba (IMIBIC)/University of Cordoba (UCO), 14002 Córdoba, Spain; 7 Department of Immunology, Reina Sofia University Hospital, 14002 Córdoba, Spain; rafael.gonzalez.sspa@juntadeandalucia.es

**Keywords:** hypoxia, angiogenesis, inflammation, coronary sinus, chronic total occlusion, percutaneous coronary intervention

## Abstract

**Background/Objectives**: The cardiac hypoxia- and inflammation-associated processes in patients with chronic coronary artery disease remain unknown. The coronary sinus (CS) can be used to explore changes in cardiac microenvironment. This study sought to evaluate acute changes in the CS concentration of hypoxia and inflammation-associated biomarkers after the percutaneous revascularization of chronic total occlusions (CTO-PCI). Additionally, we explored changes in systemic inflammation and the potential of CS biomarkers to predict left ventricular ejection fraction (LVEF) improvement on follow-up. **Methods**: Thirty-three patients undergoing CTO-PCI were included. Samples from CS were collected before and after the revascularization. Twenty-six protein biomarkers associated with hypoxia and inflammation were measured using proximity extension assay technology. Systemic inflammation markers and LVEF on cardiac magnetic resonance imaging were assessed at baseline and 6-month follow-up. **Results**: CTO-PCI yielded a significant decrease in the concentration of CS pro-angiogenic biomarkers (angiopoietin-1, vascular endothelial growth factors). In addition, there was a significant increase in the anti-inflammatory biomarker interleukin-10 and a decrease in several pro-inflammatory biomarkers like interleukin-1β. The acute response in cardiac microenvironment was followed by a mid-term reduction in systemic inflammatory markers, particularly high-sensitivity C-reactive protein. Notably, interleukin-10 showed good performance to identify patients achieving LVEF improvement on follow-up in our cohort. **Conclusions**: Our results suggest that CTO-PCI might attenuate cardiac hypoxic and inflammatory stress. These exploratory findings warrant confirmation in larger, controlled studies.

## 1. Introduction

Chronic total occlusions (CTOs) are common findings in patients with coronary artery disease (CAD), reaching a prevalence of up 15–25% and conferring worse prognosis [1]. There is current controversy about the clinical and prognostic impact of percutaneous coronary intervention of CTO (CTO-PCI). Some observational evidence reported favorable outcomes of CTO-PCI in terms of major adverse cardiovascular events (MACEs) [2], whereas the largest randomized controlled trial so far did not demonstrate such benefit [3].

The cardiac microenvironment (CmE) is composed of different types of cells in continuous interplay: endothelial cells, cardiomyocytes, myofibroblasts, and immune cells [4]. The deprivation of oxygen supply in the CmE following an acute myocardial infarction (MI) triggers an inflammatory response, with the release of angiogenic factors and chemokines [5], followed by a reparative response leaded by myofibroblasts and anti-inflammatory interleukins [6]. The presence of CTO constitutes an in vivo model of chronic myocardial ischemia, where the extent of the adaptative response to hypoxia and the modulation of inflammatory stress remain unknown.

Coronary sinus (CS) is the major cardiac vein that collects blood primarily from left ventricle myocardium and returns it to the right atrium. This structure has been broadly used in cardiovascular research to explore the CmE [7]. Even though some observational studies have evaluated the CS concentration of pro-angiogenic and pro-inflammatory biomarkers in patients with CAD [8,9], there is a lack of robust data about the hypoxic and inflammatory stress in CmE of patients with CTO.

This study sought to evaluate acute changes in the concentration of hypoxia- and inflammation-associated biomarkers from CS blood of patients undergoing CTO-PCI, to characterize their dynamics following revascularization. In addition, we explored changes in systemic inflammation markers on follow-up and the potential of CS biomarkers to predict clinically relevant improvement of left ventricular ejection fraction (LVEF).

## 2. Materials and Methods

### 2.1. Study Design and Population

**Figure 1:**
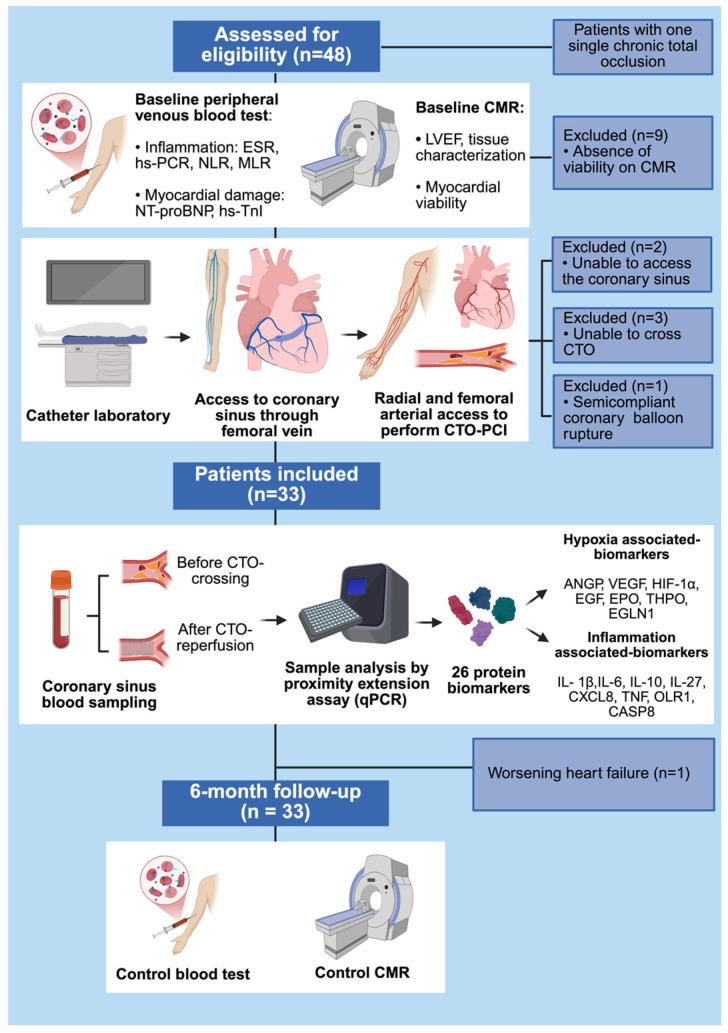
Study design and flowchart. Forty-eight patients were screened and assessed for eligibility. Of them, 15 individuals were not enrolled in the study because they did not satisfy the inclusion criteria or met the exclusion criteria. Finally, 33 patients with demonstrated myocardial viability on cardiac magnetic resonance imaging (CMR), who underwent successful percutaneous coronary intervention of chronic total occlusion (CTO-PCI), were included. During CTO-PCI, 26 protein biomarkers associated with hypoxia and inflammation were measured in coronary sinus (CS) blood using proximity extension assay technology. The most important CS biomarkers are depicted. Systemic inflammation- and myocardial-damage-associated markers were assessed at baseline and 6-month follow-up. CMR was performed at baseline and 6-month follow-up. All patients (n = 33 patients) completed the 6-month follow-up period, and one patient required hospitalization for worsening heart failure. ANGP = angiopoietin; CASP8 = caspase 8; CMR = cardiac magnetic resonance imaging; CS = coronary sinus; CTO = chronic total occlusion; CXCL = chemokine (C-X-C motif) ligand 8; EGF = endothelial growth factor; EGLN1 = egg-laying defective nine 1; EPO = erythropoietin; ESR = erythrocyte sedimentation rate; HIF-1α = hypoxia-inducible factor 1-alpha; hsCRP = high-sensitivity C-reactive protein; hsTnI = high-sensitivity troponin-I; IL = interleukin (IL-1β, IL-6, IL-10, IL-27); LVEF = left ventricular ejection fraction; MLR = monocyte-to-lymphocyte ratio; NLR = neutrophil-to-lymphocyte ratio; NT-proBNP = N-terminal pro-B-type natriuretic peptide; OLR1 = oxidized low-density lipoprotein receptor 1; PCI = percutaneous coronary intervention; qPCR = quantitative polymerase chain reaction; THPO = thrombopoietin; TNF = tumor necrosis factor; VEGF = vascular endothelial growth factor.

This is a prospective and observational study conducted at Reina Sofia University Hospital (Córdoba, Spain). Patients’ enrollment started in August 2023 and finalized in January 2025. All patients provided written informed consent before enrolment. The inclusion criteria were as follows: (i) patient aged 18–80 years; (ii) presence of one single CTO located on proximal or mid segment of one of the three main coronary vessels; (iii) demonstrated myocardial viability by cardiac magnetic resonance imaging (CMR); (iv) successful and complete CTO-PCI. The exclusion criteria were as follows: (i) acute MI three months before CTO-PCI; (ii) PCI of other coronary lesions different from CTO during the revascularization procedure; (iii) intracoronary infusion of nitroglycerine, adrenaline, or adenosine between the collection of CS samples; (iv) incomplete CTO-PCI; (v) systemic inflammatory disorders; (vi) ongoing malignant disorders. After enrolment, peripheral blood test and CMR were performed. Patients underwent CTO-PCI with CS blood sampling conducted immediately before and after the revascularization. The follow-up period was 6 months, and control blood test and CMR were performed. The study design and flowchart are depicted in Figure 1.

### 2.2. Definitions

CTO was defined as an angiographically proven anterograde flow obstruction of a coronary artery, with Thrombolysis In Myocardial Infarction (TIMI) flow grade 0, known, or suspected to have lasted >3 months based on the patient’s history [10]. Successful revascularization was defined as angiographic residual stenosis <20% and TIMI flow grade 3 after CTO-PCI. MI was defined following the universal definition endorsed by the European Society of Cardiology [11]. Myocardial viability was defined, following standards, as the presence of late gadolinium enhancement (LGE) by CMR in less than 50% of myocardial wall thickness in the territory subtended by CTO. The presence of LGE ≥50% of myocardial wall at any point of the CTO territory was considered as the absence of viability in terms of eligibility. Clinically relevant improvement of LVEF was defined as an increase of ≥5 absolute points on CMR.

### 2.3. Measurements and Procedures

#### 2.3.1. Peripheral Blood Sample Collection and Analysis

Peripheral venous blood samples were collected early in the morning after an 8 h fasting period. They were immediately transferred to the laboratory and processed following standard methods. Blood count, coagulation profile, kidney function, liver function, and lipid profile were assessed. Systemic inflammatory markers included high-sensitivity C-reactive protein (hs-CRP), neutrophil-to-lymphocyte ratio (NLR), monocyte-to-lymphocyte ratio (MLR), and erythrocyte sedimentation rate (ESR). In addition, myocardial damage-associated biomarkers N-terminal pro-B-type natriuretic peptide (NT-proBNP) and high-sensitivity troponin I (hs-TnI) were assessed. Changes from baseline in peripheral sampling were evaluated 6 months after CTO-PCI. The peripheral sampling was specifically timed to assess the stabilization of systemic chronic low-grade inflammation after the resolution of the initial response triggered by CTO-PCI.

#### 2.3.2. Cardiac Magnetic Resonance

CMR studies were performed on a 1.5-T Siemens Magnetom Aera scanner (Siemens Healthineers, Erlangen, Germany). The standard protocol included left ventricular long-axis, short-axis and three-chamber cine sequences, measurement of left ventricular volume, and calculation of LVEF. T1 and T2 mapping, LGE, and extracellular volume fraction (ECV) [12] were also assessed. Image post-processing and quantification were carried out using Syngo.via software (version VB80; Siemens Healthineers, Germany). Analysis and interpretation of all imaging data were blinded to the dynamics of CS biomarkers. Changes from baseline in CMR were evaluated 6 months after CTO-PCI.

#### 2.3.3. Percutaneous Coronary Intervention

All procedures were performed in a dedicated cardiac catheterization laboratory (Azurion 7 M12, Philips Healthcare, Best, The Netherlands) by experienced interventional cardiologists. Arterial radial access and arterial femoral access were obtained, to perform high-quality, simultaneous, dual coronary angiography. Two simultaneous pressure-monitoring systems were established. The materials and strategies used for CTO-crossing and angioplasty followed the standards of practice [13]. Unfractionated heparin (UFH) was administered before sample collection to maintain an activated clotting time of 250 to 350 s in all patients. The intracoronary infusion of nitroglycerine, adrenaline, or adenosine was strictly prohibited between sequential CS samplings. For those patients who were not on dual antiplatelet therapy, the loading dose of the second antiaggregant (clopidogrel or ticagrelor) was given the night before CTO-PCI and not during the procedure.

#### 2.3.4. Coronary Sinus Sample Collection

A venous femoral access was obtained and a multiperforated, BLK-curved, 5-frame diagnostic catheter (RadifocusTM OptitorqueTM, Terumo Corp., Tokyo, Japan) was positioned in the CS. The procedure was guided by radioscopy. Blood samples from CS were collected during CTO-PCI in two moments: at baseline, before CTO-crossing (t0), and just after CTO revascularization (t1), when achieving TIMI flow grade 3 in the previously occluded artery. Twenty milliliters of CS blood were extracted in tubes enriched with ethylenediaminetetraacetic acid. They were immediately transferred to the laboratory and centrifuged for 10 min at 3500 rpm to obtain the plasma, which was then aliquoted and stored at −80 °C until analysis. The immediate post-revascularization sampling was prioritized to capture the theoretical peak biomarker release and to isolate cardiac-specific dynamics from slower systemic metabolic shifts. Furthermore, from a safety perspective, maintaining the CS catheter after CTO-PCI would unnecessarily increase the risk of complications, such as venous thrombosis or perforation. This approach aligns with previous studies on cardiac biomarkers [9,14].

#### 2.3.5. Proximity Extension Assay

Samples from CS were subjected to targeted proteomic analysis using the Olink^®^ Flex platform (Olink Proteomics AB, Uppsala, Sweden). It is a customizable multiplex protein detection system based on proximity extension assay technology, where each protein is detected using a matched pair of antibodies conjugated to a unique DNA oligonucleotide. Upon simultaneous binding to their target protein, the oligonucleotides hybridize and can be extended by a DNA polymerase. The extension product is subsequently quantified via high-throughput quantitative polymerase chain reaction. The assay was carried out in collaboration with Cobiomic Bioscience S.L (Cordoba, Spain), who developed and validated a custom flex panel including 26 proteins, 13 of which were hypoxia-associated, and 13 were inflammation-associated. Protein concentrations were estimated in absolute terms (pg/mL). The described methodology has been previously validated in observational studies [15,16]. Detailed information about calibration/normalization, batch/run effects handling, replicate strategy, and quality control metrics is provided in the Supplementary Material.

### 2.4. Statistical Analysis

Data were expressed as absolute and percentage in the case of qualitative variables. Quantitative variables were expressed as mean ± standard deviation or median and interquartile range, depending on their distribution. Normality was assessed using the Shapiro–Wilk test and Q–Q plots. Pre–post differences in the concentration of biomarkers were analyzed using paired Student’s *t*-test or Wilcoxon signed-rank test, as appropriate. Values below or above the assay detection limits of CS biomarkers were imputed by assigning the lower or upper limit of detection, respectively. Multivariable linear mixed models were used to adjust pre–post differences for relevant covariates. Additionally, heatmap plots were generated based on Spearman’s correlation coefficients (ρ) to explore the direction of change and strength of association among biomarkers. False discovery rates (FDRs) were controlled using the Benjamini–Hochberg procedure. Receiver operating characteristic (ROC) curves were constructed to evaluate the ability of individual biomarkers to predict a clinically meaningful improvement in LVEF. The area under the curve (AUC) and its 95% confidence intervals (CI) were calculated using the DeLong method. For the biomarker demonstrating the highest discriminative performance, interleukin-10 (IL-10), an additional internal validation was performed using a bootstrapping procedure with 2000 iterations to derive 95% CI for the AUC. The optimal cutoff point was determined by the Youden Index, with its corresponding sensitivity and specificity CI estimated via the Wilson–Brown method. All statistical analyses were performed using R (version 4.3.2; R Foundation for Statistical Computing, Vienna, Austria) and GraphPad Prism (version 10.6; GraphPad Software, San Diego, CA, USA).

## 3. Results

### 3.1. The Characteristics of the Participants

A total of 33 patients with a mean age of 65.4 ± 5.9 years were included. Of them, 28 (84.8%) were men. Baseline characteristics are summarized in Table 1. Of note, 12 patients (36.4%) had diabetes mellitus, and 12 (36.4%) had obesity. The mean LVEF by CMR was 51.1 ± 10%, and 5 (15.1%) patients had LVEF < 40%, with 3 of them showing signs or symptoms of heart failure (HF). Regarding laboratory parameters, the median hsCRP was 3.5 (1.5–4.9) mg/L. Most patients had previous history of CAD (18, 54.5%), and the primary indication for CTO-PCI was persistent angina despite optimal medical treatment (24, 72.7%). The main location of CTO was right coronary artery (20, 60.2% of patients), and the mean procedure time was 130 ± 55 min. There were no major complications related to CTO-PCI (cardiac tamponade, acute stent thrombosis, MI or patient’s death).

### 3.2. Acute Changes in Hypoxia-Associated Biomarkers After CTO-PCI

**Figure 2:**
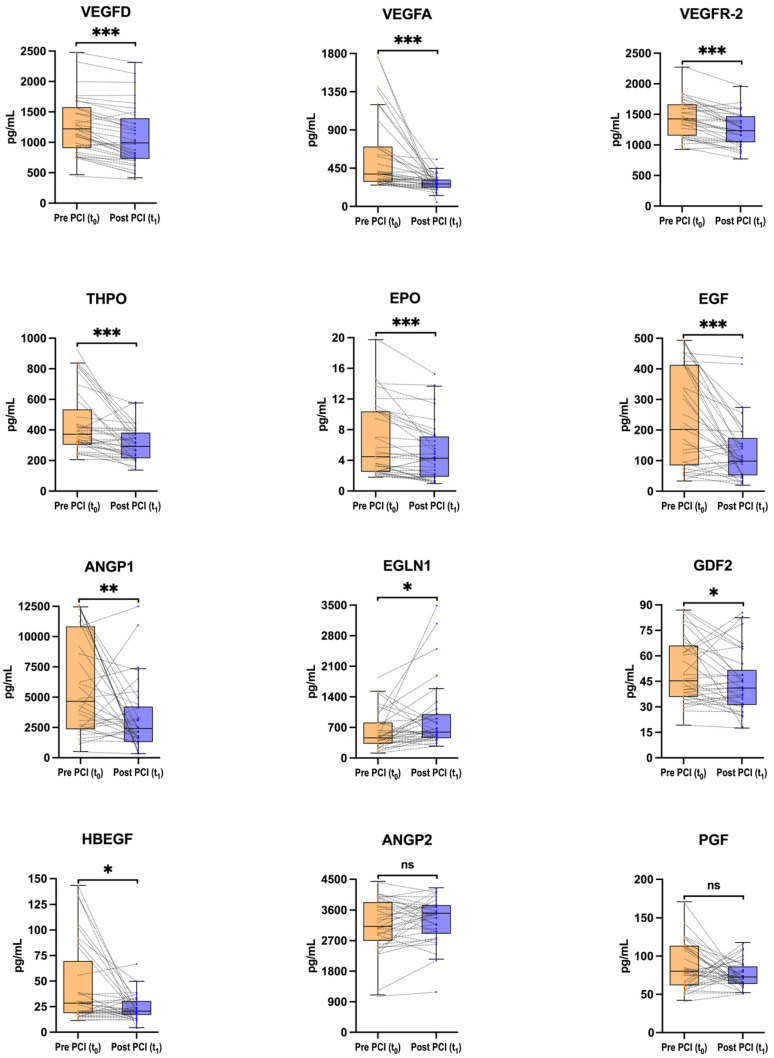
Changes in hypoxia-associated biomarkers from coronary sinus blood during percutaneous coronary intervention for chronic total occlusions (CTO-PCI). Pairwise changes in absolute concentrations (pg/mL) of 12 hypoxia-associated biomarkers during CTO-PCI are shown. Biomarker concentrations were measured in samples from coronary sinus blood using proximity extension assay technology (Olink Proteomics, Uppsala, Sweden), a dual-recognition immunoassay based on quantitative polymerase chain reaction. Samples were obtained in two moments: at baseline, before CTO-crossing (t0) (n = 33 patients), and post CTO revascularization (t1) (n = 33 patients), when achieving a Thrombolysis In Myocardial Infarction flow grade 3 in the previously occluded artery. The median time between t0 and t1 was 98 (56–120.5) min. The mean procedural time was 130 ± 55 min. Biomarkers are ordered by statistical significance level. Box plots display median and interquartile range (IQR); whiskers indicate 1.5×IQR. Dots represent individual patients; dotted lines connect paired measurements. Two-tailed paired test according to variable distribution (Student’s t or Wilcoxon test). * *p* < 0.05, ** *p* < 0.01, *** *p* < 0.001, ns = not significant, according to raw *p*-values. Full descriptive data, raw and FDR-adjusted *p*-values for paired tests, and average pre–post differences are provided in Supplementary Table S1. ANGP = angiopoietin (ANGP1, ANGP2); CTO = chronic total occlusion; EGF = endothelial growth factor; EGLN1 = egg-laying defective nine 1; EPO = erythropoietin; GDF2 = growth differentiation factor 2; HBEGF = heparin-binding EG-like growth factor; IQR = interquartile range; PGF = placenta growth factor; THPO = thrombopoietin; VEGF = vascular endothelial growth factor (VEGFA, VEGFD); VEGFR-2 = vascular endothelial growth factor receptor-2.

The Olink^®^ Flex platform showed good performance to detect 12 of 13 biomarkers, only with hypoxia-inducible factor 1-alpha (HIF-1α) out of the limits of detection (91% of samples below the range). Changes in hypoxia-associated CS biomarkers after CTO-PCI are depicted in Figure 2. Full descriptive data, including detection ranges, and results from pairwise tests and multivariable analysis are provided in Supplementary Tables S1 and S2.

There was a significant decrease in classic pro-angiogenic biomarkers following CTO-PCI: vascular endothelial growth factor A (VEGFA) [382 (291–704) pg/mL baseline (t0) vs. 264.3 (220–316) pg/mL post CTO-PCI (t1), *p* < 0.001, FDR-adj *p* < 0.001], vascular endothelial growth factor receptor-2 (VEGFR-2) (1440 ± 301.6 pg/mL vs. 1253 ± 267.4 pg/mL, *p* < 0.001, FDR-adj *p* < 0.001) and angiopoietin-1 (ANGP1) [4658 (2493–10,628) pg/mL vs. 2483 (1323–4076) pg/mL, *p* = 0.006, FDR-adj *p* = 0.015]. Other pro-angiogenic biomarkers, like vascular endothelial growth factor D (VEGFD), erythropoietin (EPO), thrombopoietin (THPO), and endothelial growth factor (EGF) also exhibited a significant decrease after CTO-PCI. Both heparin-binding EGF-like growth factor (HBEGF) and growth differentiation factor 2 (GDF2, also known as bone morphogenetic protein 9) showed downward trends with *p*-values at the borderline of statistical significance. By contrast, the concentration of the prolyl-hydroxylase egg-laying defective nine 1 (EGLN1) significantly increased in CS blood after CTO-PCI [462 (328–812) pg/mL vs. 595 (458–1003) pg/mL, *p* = 0.017, FDR-adj *p* = 0.033]. All changes mentioned above remained significant after multivariable analysis adjusting for sex, age, baseline (t0) concentration of the biomarker, baseline LVEF, diabetes mellitus, estimated glomerular filtration rate, and key procedural variables (procedural time, CTO location, collateral grade according to the Rentrop classification, approach for CTO-crossing).

Baseline (t0) concentrations of ANGP1, VEGFA, EGF, and HBEGF were correlated (ρ > 0.75, FDR-adj *p* < 0.001), as well as their decrease after CTO-PCI (ρ > 0.65, FDR-adj *p* < 0.01). In addition, the increase in EGLN1 was correlated with the decrease in caspase-8 (CASP8) (ρ = −0.90, FDR-adj *p* < 0.001). Correlation coefficients and FDR-adj *p*-values of hypoxia-associated biomarkers are provided in Supplementary Table S3.

### 3.3. Acute Changes in Inflammation-Associated Biomarkers After CTO-PCI

**Figure 3:**
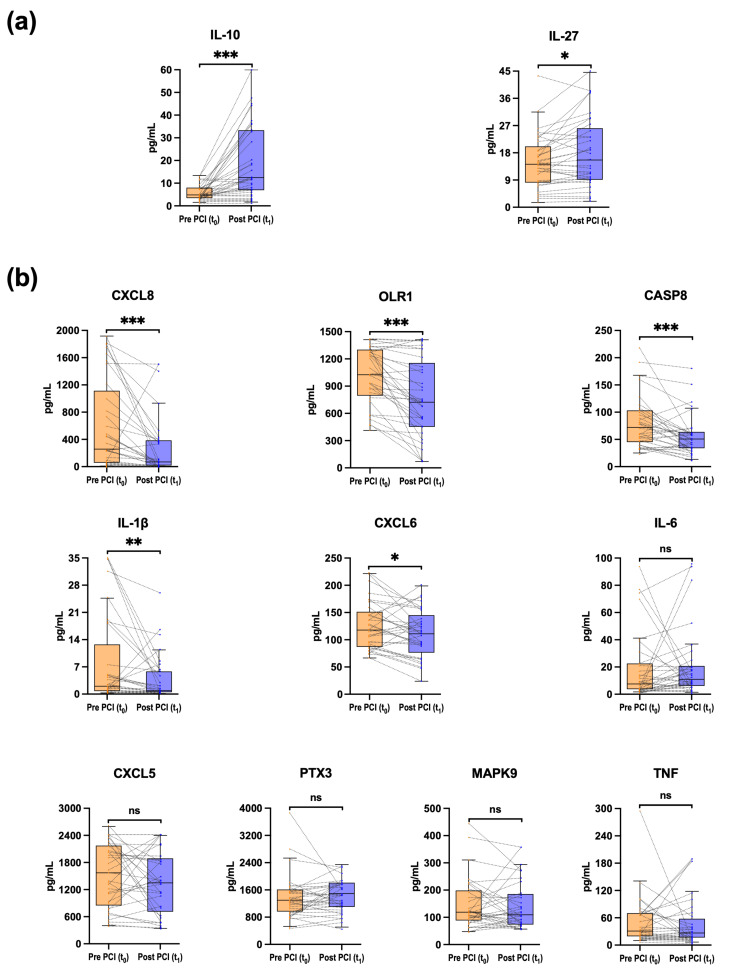
Changes in inflammation-associated biomarkers from coronary sinus blood during percutaneous coronary intervention for chronic total occlusions (CTO-PCI). Pairwise changes in absolute concentrations (pg/mL) of 12 inflammation-associated biomarkers during CTO-PCI are shown. (**a**) Anti-inflammatory biomarkers and (**b**) pro-inflammatory biomarkers. Biomarker concentrations were measured in samples from coronary sinus blood using proximity extension assay technology (Olink Proteomics, Uppsala, Sweden), a dual-recognition immunoassay based on quantitative polymerase chain reaction. Samples were obtained in two moments: at baseline, before CTO-crossing (t0) (n = 33 patients), and post CTO revascularization (t1) (n = 33 patients), when achieving a Thrombolysis In Myocardial Infarction flow grade 3 in the previously occluded artery. The median time between t0 and t1 was 98 (56–120.5) min. The mean procedural time was 130 ± 55 min. Biomarkers are ordered by statistical significance level. Box plots display median and interquartile range (IQR), whiskers indicate 1.5xIQR. Dots represent individual patients; dotted lines connect paired measurements. Two-tailed Wilcoxon signed-rank test. * *p* < 0.05, ** *p* < 0.01, *** *p* < 0.001, ns = not significant, according to raw *p*-values. Full descriptive data, raw and FDR-adjusted *p*-values for paired tests, and average pre–post differences are provided in Supplementary Table S1. CASP8 = caspase 8; CTO = chronic total occlusion; CXCL = chemokine (C-X-C motif) ligand (CXCL5, CXCL6, CXCL8); IL = interleukin (IL-1β, IL-6, IL-10, IL-27); IQR = interquartile range; MAPK9 = mitogen-activated protein kinase 9; OLR1 = oxidized low-density lipoprotein receptor 1; PTX3 = pentraxin 3; TNF = tumor necrosis factor.

The Olink^®^ Flex platform showed good performance to detect 12 of 13 biomarkers, only with tumor necrosis factor ligand superfamily member 12 out of the limits of detection (87% of samples above the range). Changes in inflammation-associated CS biomarkers during CTO-PCI are depicted in Figure 3. Full descriptive data, including detection ranges, and results from pairwise tests and multivariable analysis are provided in Supplementary Tables S1 and S2.

The concentration of the anti-inflammatory biomarker IL-10 significantly increased after CTO-PCI [4.8 (3.5–8) pg/mL vs. 12.5 (6.9–33.3) pg/mL, *p* < 0.001, FDR-adj *p* < 0.001]. The same response was observed for interleukin-27 (IL-27) [14.2 (8.1–20.1) pg/mL vs. 15.6 (9.3–26.1) pg/mL, *p* = 0.010, FDR-adj *p* = 0.022]. By contrast, the concentration of some pro-inflammatory biomarkers significantly decreased: interleukin-1β (IL-1β) [2 (0.8–12.8) pg/mL vs. 0.9 (0.5–5.8) pg/mL, *p* = 0.002, FDR-adj *p* = 0.005], and the chemokine (C-X-C motif) ligand 8 (CXCL8, also known as interleukin-8) [257.5 (58–1114) pg/mL vs. 70 (17.2–386.7) pg/mL, *p* < 0.001, FDR-adj *p* < 0.001], with the chemokine (C-X-C motif) ligand 6 (CXCL6) showing *p*-values at the borderline of statistical significance. Furthermore, the concentration of CASP8 and oxidized low-density lipoprotein receptor 1 (OLR1) significantly decreased following the revascularization. After multivariable adjustment, all changes remained significant.

**Figure 4:**
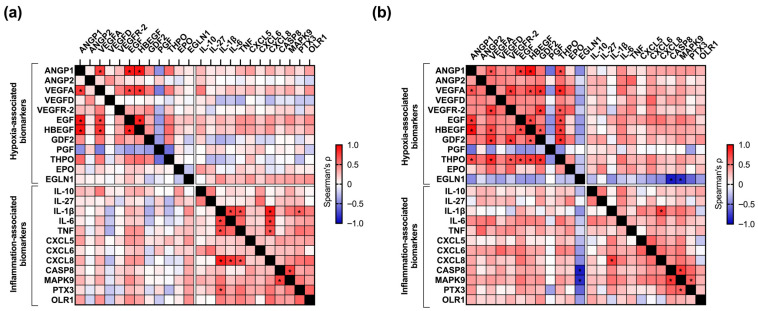
Correlation network of coronary sinus biomarkers. (**a**) Heatmap of baseline (t0) concentration of coronary sinus (CS) biomarkers, before percutaneous coronary intervention of chronic total occlusion (CTO-PCI) (n = 33 patients) and (**b**) heatmap of changes (Δ post–pre-CTO-PCI) in the concentration of CS biomarkers (n = 33 patients). Heatmaps display Spearman’s correlation coefficients (ρ) among biomarkers, ordered by functional cluster (hypoxia-associated and inflammation-associated). Color scale indicates the strength and direction of the correlation (red = positive, blue = negative). Asterisks (*) identify correlations with |ρ| ≥ 0.65 and FDR-adjusted *p* < 0.01. Coefficients and FDR-adjusted *p*-values are provided in Supplementary Table S3 for hypoxia-associated and in Supplementary Table S4 for inflammation-associated biomarkers. The concentration of CS biomarkers was measured using proximity extension assay technology (Olink Proteomics, Uppsala, Sweden), a dual-recognition immunoassay based on quantitative polymerase chain reaction. Samples were obtained in two moments: at baseline, before CTO-crossing (t0) (n = 33 patients), and post CTO revascularization (t1) (n = 33 patients), when achieving a Thrombolysis In Myocardial Infarction flow grade 3 in the previously occluded artery. The median time between t0 and t1 was 98 (56–120.5) min. The mean procedural time was 130 ± 55 min. ANGP = angiopoietin (ANGP1, ANGP2); CASP8 = caspase 8; CS = coronary sinus; CTO = chronic total occlusion; CXCL = chemokine (C-X-C motif) ligand (CXCL5, CXCL6, CXCL8); EGF = endothelial growth factor; EGLN1 = egg-laying defective nine 1; EPO = erythropoietin; FDR = false discovery rate; GDF2 = growth differentiation factor 2; HBEGF = heparin-binding EGF-like growth factor; IL = interleukin (IL-1β, IL-6, IL-10, IL-27); MAPK9 = mitogen-activated protein kinase 9; OLR1 = oxidized low-density lipoprotein receptor 1; PCI = percutaneous coronary intervention; PGF = placenta growth factor; PTX3 = pentraxin 3; THPO = thrombopoietin; TNF = tumor necrosis factor; VEGF = vascular endothelial growth factor (VEGFA, VEGFD); VEGFR-2 = vascular endothelial growth factor receptor-2.

The correlation analysis showed association between baseline (t0) concentration of IL-1β, CXCL8, interleukin-6 (IL-6), and tumor necrosis factor (TNF) (ρ > 0.65, FDR-adj *p* < 0.01). The decrease in the concentration of IL-1β and CXCL8 after CTO-PCI was also correlated (ρ > 0.65, FDR-adj *p* < 0.01). The correlation network of CS biomarkers is depicted in Figure 4. Correlation coefficients and FDR-adj *p*-values of inflammation-associated biomarkers are provided in Supplementary Table S4.

### 3.4. Mid-Term Changes in Systemic Inflammation and Myocardial Damage-Associated Markers After CTO-PCI

**Figure 5:**
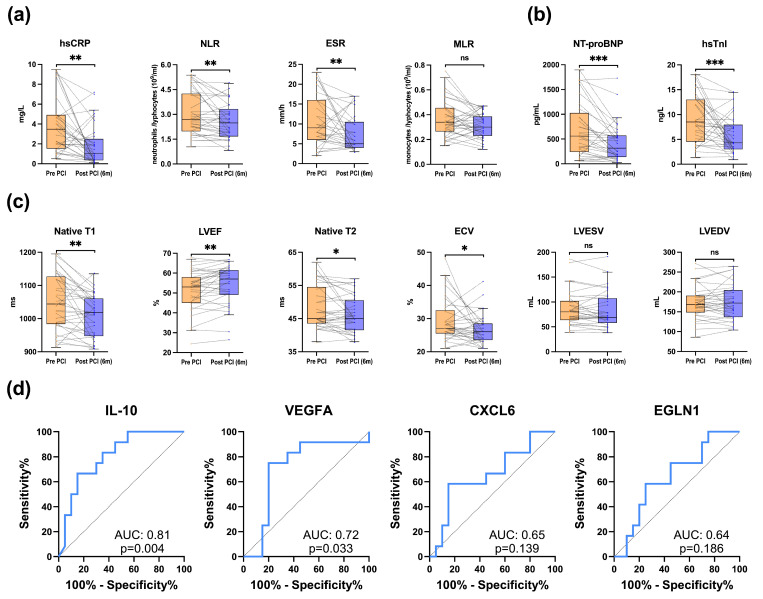
Changes in systemic markers and cardiac magnetic resonance imaging parameters at 6-month follow-up. (**a**) Pairwise changes in systemic inflammation-associated markers measured in peripheral venous blood after percutaneous coronary intervention of chronic total occlusion (CTO-PCI), at 6-month follow-up (n = 33 patients). (**b**) Pairwise changes in myocardial damage-associated markers measured in peripheral venous blood after CTO-PCI, at 6-month follow-up (n = 33 patients). (**c**) Pairwise changes in cardiac magnetic resonance imaging (CMR) parameters (n = 33 patients) after CTO-PCI, at 6-month follow-up. (**d**) Receiver operating characteristic (ROC) curves of the baseline (t0) concentration of coronary sinus (CS) biomarkers with the highest discriminative performance for predicting a clinically relevant improvement of LVEF (≥5 absolute points) after CTO-PCI, at 6-month follow-up CMR (n = 33 patients). The concentration of CS biomarkers was measured using proximity extension assay technology (Olink Proteomics, Uppsala, Sweden), a dual-recognition immunoassay based on quantitative polymerase chain reaction. Box plots depict median and interquartile range (IQR), whiskers 1.5xIQR. Dots represent individual patients, and thin grey dotted lines connect paired measurements. (**a**–**c**) Two-tailed Student’s *t*-test or Wilcoxon test. * *p* < 0.05, ** *p* < 0.01, *** *p* < 0.001, ns = not significant, according to raw *p*-values. Full descriptive data and results from pairwise tests are provided in Supplementary Table S5. (**d**) Sensitivity is plotted on the y-axis and 100-specificity on the x-axis. For each biomarker, the AUC with its raw *p*-value is shown on the plot; higher AUC reflect better discrimination. The AUC and its 95% confidence interval were calculated using the DeLong method. ROC curves for the entire panel of CS biomarkers are provided in Supplementary Figure S1. Detailed data from ROC analysis is provided in Supplementary Table S6. AUC = area under the curve; CMR = cardiac magnetic resonance imaging; CS = coronary sinus; CTO = chronic total occlusion; CXCL6 = chemokine (C-X-C motif) ligand 6; ECV = extracellular volume; EGLN1 = egg-laying defective nine 1; ESR = erythrocyte sedimentation rate; hsCRP = high-sensitivity C-reactive protein; hsTnI = high-sensitivity troponin-I; IL-10 = interleukin 10; IQR = interquartile range; LVEDV = left ventricular end diastolic volume; LVEF = left ventricular ejection fraction; LVESV = left ventricular end systolic volume; NLR = neutrophil-to-lymphocyte ratio; NT-proBNP = N-terminal pro-B-type natriuretic peptide; MLR = monocyte-to-lymphocyte ratio; PCI = percutaneous coronary intervention; ROC = receiver operating characteristic; VEGFA = vascular endothelial growth factor A.

A significant decrease in systemic inflammation markers hsCRP [3.5 (1.5–4.9) mg/L vs. 1 (0.3–2.5) mg/L, *p* = 0.001, FDR-adj *p* = 0.016], NLR [2.7 (2–4.2) vs. 2.4 (1.6–3.3), *p* = 0.004, FDR-adj *p* = 0.028] and ESR [9 (6–16) mm/h vs. 5 (3–10.5) mm/h, *p* = 0.005, FDR-adj *p* = 0.036] was observed at 6-month follow-up, whereas MLR showed downward non-significant trend. In addition, there was a significant decrease in the levels of NT-proBNP [560 (241–1027) pg/mL vs. 318 (137–572) pg/mL, *p* < 0.001, FDR-adj *p* = 0.007] and hsTnI [9.4 ± 4.94 ng/L vs. 5.93 ± 3.91 ng/L, *p* < 0.001, FDR-adj *p* = 0.010]. Changes in systemic markers after CTO-PCI are depicted in Figure 5. Full descriptive data and results from pairwise tests are provided in Supplementary Table S5.

### 3.5. Coronary Sinus Biomarkers and Mid-Term Changes in Cardiac Magnetic Resonance Imaging After CTO-PCI

There was a significant increase in mean LVEF by CMR 6 months after CTO-PCI [51.15 ± 10% vs. 53.58 ± 10.2%, *p* = 0.007, FDR-adj *p* = 0.028], with 12 of 33 patients experiencing a clinically relevant improvement, in the absence of significant changes in left ventricular volumes. A significant reduction in native T1 was observed [1050 ± 84.77 ms vs. 1010 ± 62.28 ms, *p* = 0.002, FDR-adj *p* = 0.016], while reductions in native T2 and ECV did not remain significant after FDR adjustments. Changes in CMR parameters on follow-up are depicted in Figure 5. Full descriptive data and results from pairwise tests are provided in Supplementary Table S5.

Among the CS biomarkers, IL-10 demonstrated the highest discriminative performance to identify patients achieving clinically relevant improvement in LVEF on follow-up CMR. The ROC curve for baseline (t0) IL-10 concentration yielded an AUC of 0.81 (95% CI: 0.58 to 0.94, derived from 2.000 bootstrap resamples; *p*= 0.004). A cut-off point of >5.45 pg/mL for baseline (t0) IL-10 concentration provided a likelihood ratio for LVEF improvement of 4.44, with a sensitivity of 67% (95% CI: 39% to 86%) and specificity of 85% (95% CI: 64% to 95%). ROC curves for IL-10, VEGFA, CXCL6, and EGLN1 are depicted in Figure 5, while the rest are provided in Supplementary Figure S1. Detailed data from ROC analysis is provided in Supplementary Table S6. Supplementary Table S7 details the sensitivity and specificity values across different IL-10 cut-off points.

## 4. Discussion

In this study, we evaluated acute changes in cardiac hypoxia- and inflammation-associated biomarkers during CTO-PCI. The main findings were the following: (a) There was a decrease in pro-angiogenic and pro-inflammatory biomarkers; (b) There was an increase in anti-inflammatory biomarkers; (c) There was a decrease in systemic inflammatory markers at 6-month follow-up; and (d) IL-10 showed good performance to identify patients achieving clinically relevant LVEF improvement at 6-month follow-up.

### 4.1. CTO-PCI Modulates the Hypoxic and Inflammatory Stress in CmE: Mechanistic Insights

The restoration of oxygen supply to the ischemic myocardium through CTO-PCI resulted in an acute decrease in VEGF. This family of growth factors are key regulators of angiogenesis in patients with CAD [8]. In our study, VEGFA, VEGFD, and VEGFR-2 significantly decreased, and the strongest correlation was found between the change in VEGFA and VEGFR-2, which might be related to their high affinity [17]. A significant decrease was also observed in ANGP1, showing a moderate-to-strong correlation with VEGFA. It might stem from the pivotal role of ANGP1 signaling in angiogenesis: it enhances the transcription of VEGFA mRNA and promotes the stability of neo vessels [18]. The lack of a significant reduction in angiopoietin-2 (ANGP2) could be attributable to its context-dependent activity, with a less consistent angiogenic profile than ANGP1 and the potential to promote vascular destabilization [19]. Other biomarkers that also showed a significant reduction after the revascularization—including EGF, THPO, EPO, GDF2, and HBEGF—are likewise implicated in angiogenic pathways in both physiological and pathological settings [20,21,22]. The overall reduction observed across the group of pro-angiogenic biomarkers in CS, with a moderate-to-strong correlation among them, might stem from an attenuation of hypoxic stress within the CmE after CTO-PCI.

Interestingly, EGLN1 significantly increased after CTO-PCI, which might reflect the increase in oxygen availability in CmE. EGLN1 enhances the degradation of HIF-1α under normoxic conditions, playing the role of intracellular oxygen-sensor [23]. Furthermore, the increase in EGLN1 levels was strongly correlated with the decrease in CASP8. CASP8 promotes apoptosis via receptor-interacting protein kinase 1 (RIPK1) [24], and some observational evidence has reported a key role of the EGLN1 pathway in suppressing RIPK1 under normoxic conditions to promote cell survival [25]. Therefore, our findings appear to be consistent with a reduction in hypoxic stress and a subsequent decrease in apoptotic pathways in CmE after CTO-PCI. The correlations among other biomarkers from different clusters were weaker and nonsignificant, which might be explained by the fact that inflammation and hypoxia-associated biomarkers are regulated with different temporal dynamics.

Regarding pro-inflammatory biomarkers, baseline (t0) concentrations of IL-1β, IL-6, TNF, and CXCL8 were moderate-to-strongly correlated, which could be linked to the fact that they share common inflammatory pathways [26]. CTO-PCI yielded a significant decrease in IL-1β, CXCL8, and OLR1, which play a crucial role in the local inflammatory responses in atherosclerotic plaques [26,27]. Therefore, their reduction suggests an attenuation in the inflammatory stress in CmE. Similarly, there was a mid-term decrease in systemic hsCRP, a marker of enhanced trained immunity in patients with CAD [28], suggesting an attenuation of the systemic chronic pro-inflammatory stress after CTO-PCI.

IL-10 significantly increased in CS blood after CTO-PCI. This anti-inflammatory interleukin plays a protective role in CAD, contributing to the stability of the atherosclerotic plaque and promoting reparative responses after MI [29]. In addition, IL-27 concentration increased, which might be linked to its immunomodulatory ability, inducing IL-10 and promoting T-regulatory responses [30]. Thus, the rise in these biomarkers might reflect an anti-inflammatory shift in CmE after CTO-PCI. In addition, IL-10 resulted in a good discriminative performance to identify clinically relevant improvement of LVEF on follow-up. From a physiological perspective, a higher baseline (t0) concentration of IL-10 might indicate enhanced upregulation of reparative pathways in CmE, potentially increasing the probability of LVEF improvement after CTO-PCI.

### 4.2. Previous Studies Evaluating the Hypoxic and Inflammatory Stress in CmE

There is limited evidence addressing the effect of PCI on hypoxia-associated biomarkers in CmE. To date, only one observational study has simultaneously evaluated changes in the concentration of VEGF, ANGP1, and ANGP2 in CS blood following elective PCI [14]. Consistent with our findings, they reported a significant post-procedural decrease in ANGP1, although VEGF and ANGP2 levels remained unchanged. In the setting of acute MI, some studies using peripheral blood samples have shown a significant increase in VEGF [31] and a significant decrease in ANGP1 and ANGP2 [32,33]. While our results align with the observed ANGP1 reduction across both cardiac and peripheral compartments, the discrepancies in ANGP2 and VEGF dynamics likely stem from differences in clinical context (exclusion of CTO patients), sampling location, and timing. Specifically, peripheral markers in previous studies were measured days or weeks post-PCI, whereas our study captures the acute phase, potentially missing more latent temporal changes. These variations underscore the impact of non-standardized sampling protocols on biomarker kinetics.

Regarding changes in inflammation-associated biomarkers in CmE, our findings diverge from previous observational studies that reported early increases in CS concentrations of IL-1β and IL-6 after elective PCI [9,34]. Nevertheless, they did not include patients with CTO, and they used some types of stents and plaque-modification techniques that could affect the early inflammatory response after PCI. In fact, in a small observational study, the levels of IL-6 after bare metal stenting significantly increased, but they returned to baseline in 12 h [35]. Similarly, our results contrast with the evidence on ischemia-reperfusion injury in acute MI, where PCI typically triggers an increase in both cardiac and systemic inflammation [36,37,38]. These discrepancies likely reflect the distinct biological context of CTO. Unlike acute scenarios, CTO-PCI might primarily mitigate long-standing inflammatory stress rather than induce additional injury.

### 4.3. Clinical Implications and Translational Perspective

The chronic pro-inflammatory stress in CmE contributes to myocardial mass loss, impaired contractility, and subsequent HF [39]. Furthermore, this environment promotes the atherosclerotic plaque destabilization increasing the risk of MACE [40]. Consequently, the reduction in CS levels of pro-angiogenic and pro-inflammatory biomarkers observed after CTO-PCI might reflect a clinically beneficial anti-inflammatory shift. This concept aligns with the CANTOS trial, which demonstrated that targeting IL-1β reduces recurrent MACE in patients with CAD [41].

Consistently, we observed a significant decrease in systemic inflammatory markers, specifically hsCRP and NLR, at 6-month follow-up. Given their established role in predicting adverse outcomes in CAD [42,43], this sustained reduction might be relevant from the clinical perspective. Changes in systemic inflammation can be influenced by intercurrent diseases, lifestyle, or pharmacological changes. However, our cohort remained stable: 97% were on baseline statin therapy with no significant modifications during follow-up (only one patient required the addition of ezetimibe), and no intercurrent inflammatory events were reported. Furthermore, the concomitant decrease in NT-proBNP and hsTnI, which have been associated with cardiovascular mortality [44], underscores the potential clinical benefit of CTO-PCI. The changes in these myocardial damage-associated markers can also be influenced by medication and intercurrent cardiac events. Importantly, all patients with LVEF < 40% in our cohort received guideline-directed quadruple therapy at baseline. During follow-up, 2 patients required dose optimization of prognostic HF therapy, and 1 patient required diuretic treatment increase and hospitalization because of worsening HF. No further cardiac events were reported.

From a translational perspective, characterizing the hypoxic and inflammatory stress in CTO patients could help identify those at higher risk for MACE or those most likely to benefit functionally from revascularization. In our cohort, baseline (t0) CS levels of IL-10 showed a positive correlation with LVEF improvement at 6-month follow-up. This suggests that a robust baseline anti-inflammatory reserve might be essential for optimal functional recovery after chronic ischemia is relieved. Emerging imaging tools, such as coronary computed tomography angiography, have been proposed to evaluate plaque vulnerability and perivascular inflammation, potentially complementing biochemical data in future screening protocols [45]. While our findings provide encouraging exploratory evidence, these molecular signatures should be considered hypothesis-generating. The diagnostic accuracy and predictive value of these biomarkers require validation in larger, independent populations.

### 4.4. Study Limitations

Regarding the methodological framework, the observational nature of this study provides primarily associative evidence, and residual confounding factors cannot be entirely ruled out. To address this issue, we employed multivariable analyses adjusting relevant covariates, including age, sex, and baseline (t0) values of CS biomarkers—accounting for regression-to-the-mean effects. The absence of a control group is an inherent limitation due to the technical complexity and invasiveness of CTO-PCI and CS sampling. Furthermore, certain anatomical and procedural factors should be analyzed. The extent of the drained myocardial mass and the grade of collateral circulation may modulate cardiac signaling. Left anterior descending coronary artery CTO is typically associated with larger myocardial involvement and could lead to greater baseline (t0) concentrations and post-revascularization changes in CS biomarkers, whereas a higher grade of collateral circulation could attenuate them. Nevertheless, both CTO location and collateral grade were included in the multivariable linear mixed model, with the observed changes in CS biomarkers remaining statistically significant. Similarly, periprocedural medications like UFH or intracoronary vasoactive agents may modulate hypoxic and inflammatory stress. However, the administration of UFH was standardized to minimize bias, and no intracoronary vasoactive agents were allowed between CS samplings. Lastly, the absence of simultaneous peripheral sampling limits a complete separation of cardiac-specific from systemic influences.

From a statistical perspective, the limited sample size and the multiplicity of measured biomarkers underscore the exploratory nature of our findings. This framework introduces a dual statistical challenge: first, the relatively small cohort increases the risk of type II error, potentially limiting the power to detect further significant associations. Conversely, the high number of simultaneous measurements increases the probability of type I error. To mitigate the risk of false positives arising from multiple comparisons, FDR adjustments were considered during the analysis of biomarker dynamics. Nevertheless, these results should be interpreted as hypothesis-generating and require confirmation in larger, independent populations.

Finally, from a translational perspective, the clinical utility of these molecular signatures as predictors of functional recovery requires cautious interpretation. Although the baseline (t0) concentration of IL-10 demonstrated a good performance in predicting LVEF improvement, the extrapolation of these results to broader clinical practice is limited by the current cohort size. Further prospective studies are essential to establish definitive cut-off values and validate the long-term predictive accuracy. However, the study provides in vivo human data about cardiac hypoxic and inflammatory stress obtained during complex interventional procedures, which may support its clinical and translational relevance.

## 5. Conclusions

The revascularization of a CTO in patients with myocardial viability seemed to be associated with acute in vivo changes in the concentration of CS biomarkers associated with hypoxia and inflammation. Our results suggest that CTO-PCI might attenuate the cardiac hypoxic and inflammatory stress. However, given the limited sample size, the multiplicity of measurements, and the mixed nature of CS sampling, these observations are mostly exploratory and warrant confirmation in larger, controlled studies.

## Figures and Tables

**Table 1:** Baseline characteristics of the participants.

Characteristic	Patients (n = 33)
Age (years)	65.4 ± 5.9
Male sex	28 (84.8)
BMI (kg/m^2^)	26.9 ± 3.3
Obesity	12 (36.4)
Hypertension	22 (66.7)
Diabetes mellitus	12 (36.3)
Hyperlipidemia	23 (69.7)
Smoking habit	19 (57.6)
Atrial fibrillation	2 (6)
Cerebrovascular disease	3 (9.1)
Valvular heart disease	1 (3)
Heart failure	3 (9.1)
LVEF by CMR (%)	51.1 ± 10
LVEF ≥ 50	23 (69.7)
LVEF < 50	10 (30.3)
History of CAD	18 (54.5)
Stable angina	5 (15.1)
STEMI	6 (18.2)
NSTEMI	7 (21.2)
**Laboratory data**	
eGFR (mL/min/1.73 m^2^) (90–120) *	85 ± 11.4
HbA1c (%) (4–6)	6.0 (5.7–6.7)
Total cholesterol (mmol/L) (3.6–5.7)	2.4 ± 0.38
LDL-c (mmol/L) (1.3–3.4)	1.35 ± 0.23
HDL-c (mmol/L) (1.1–2.1)	1.15 ± 0.19
Triglycerides (mmol/L) (0.5–6.5)	1.21 ± 0.32
hsCRP (mg/L) (0–10)	3.5 (1.5–4.9)
ESR (mm/h) (0–15)	9 (6–16)
hsTnI (ng/L) (0–46)	9.1 ± 4.9
NT-proBNP (pg/mL) (0–125)	560 (241–1027)
**Angiographic data**	
SYNTAX ^†^	16.5 ± 6
J-CTO ^‡^	2 (2–3)
Rentrop ^¥^	2 (2–3)
Location of CTO	
LAD	10 (30.3)
LCX	3 (9.1)
RCA	20 (60.6)
Indication for CTO-PCI–	
Persistent angina	24 (72.7)
Inducible ischemia	4 (12.2)
Ventricular dysfunction (LVEF < 40)	5 (15.1)
Approach for CTO-crossing	
Antegrade only	20 (60.6)
Antegrade–retrograde	13 (39.4)
Procedure time (min)	130 ± 55
Pre–post CS sampling time	98 (56–120.5)
Fluoroscopy time (min)	32 ± 10
Contrast volume (mL)	198 ± 41
**Medical treatment**	
ACEI/ARBs	24 (72.7)
ARNI	5 (15.1)
Beta blocker	23 (69.7)
MRA	6 (18.1)
SGLT2i	8 (24.2)
CCB	8 (24.2)
Nitrates (oral/transdermic)	13 (39.4)
Statins	32 (97.0)
Ezetimibe	23 (69.7)
PCSK9i	2 (6.0)
Antiplatelet drugs	
ASA	33 (100)
P2Y12i	12 (36.3)

Qualitative variables are expressed as n (%); quantitative variables are expressed as mean ± standard deviation or median and interquartile range. * Reference values established in our central laboratory. ^†^ SYNTAX score assesses the complexity of coronary artery disease. ^‡^ J-CTO score assesses the technical complexity of the percutaneous revascularization of chronic total occlusions. ^¥^ Rentrop classification assesses the grade of collateral circulation. ACEI = angiotensin-converting enzyme inhibitor; ARB = angiotensin receptor blocker; ARNI = angiotensin receptor-neprilysin inhibitor; ASA = acetylsalicylic acid; BMI = body mass index; CAD = coronary artery disease; CCB = calcium channel blocker; CMR = cardiac magnetic resonance; CS = coronary sinus; CTO = chronic total occlusion; eGFR = estimated glomerular filtration rate; ESR = erythrocyte sedimentation rate; HbA1c = glycosylated hemoglobin; HDL-c = high-density lipoprotein cholesterol; hsCRP = high-sensitivity C-reactive protein; hsTnI = high-sensitivity troponin I; J-CTO = Japanese chronic total occlusion; LAD = left anterior descending coronary artery. LCX = left circumflex coronary artery; LDL-c = low-density lipoprotein cholesterol; LVEF = left ventricular ejection fraction; MRA = mineralocorticoid receptor antagonist. NSTEMI = non-ST-segment elevation myocardial infarction; NT-proBNP = N-terminal pro-B-type natriuretic peptide; PCI = percutaneous coronary intervention; PCSK9i = proprotein convertase subtilisin/kexin type 9 inhibitor; P2Y12i = P2Y12 receptor inhibitors; RCA = right coronary artery; SGLT2i = sodium-glucose contransporter-2 inhibitor; STEMI = ST-segment elevation myocardial infarction; SYNTAX = synergy between percutaneous coronary intervention with Taxus and cardiac surgery.

## Data Availability

The de-identified data supporting the findings of this study, as well as the analysis code used to generate the results, are available from the corresponding author upon reasonable request.

## Supplementary Materials

The following supporting information can be downloaded at https://www.mdpi.com/article/10.3390/jcm15020517/s1. Olink Flex calibration/normalization, batch/run effects handling, replicate strategy, and quality control metrics. Figure S1: ROC curves of coronary sinus biomarkers. Table S1: Descriptive statistics and paired comparisons of coronary sinus biomarkers. Table S2: Multivariable linear mixed model for changes in coronary sinus biomarkers. Table S3: Correlation matrix for hypoxia-associated biomarkers. Table S4: Correlation matrix for inflammation-associated biomarkers. Table S5: Changes in systemic biomarkers and CMR parameters 6 months after CTO-PCI. Table S6: ROC analysis of coronary sinus biomarkers for predicting left ventricular ejection fraction improvement. Table S7: Discriminative performance across different interleukin-10 cutoff points for predicting left ventricular ejection fraction improvement.

## Abbreviations

The following abbreviations are used in this manuscript:

ACEI	Angiotensin-converting enzyme inhibitor
ANGP	Angiopoietin (ANGP1, ANGP2)
ARB	Angiotensin receptor blocker
ARNI	Angiotensin receptor-neprilysin inhibitor
ASA	Acetylsalicylic acid
AUC	Area under curve
BMI	Body mass index
CAD	Coronary artery disease
CASP8	Caspase-8
CCB	Calcium channel blocker
CI	Confidence interval
CmE	Cardiac microenvironment
CMR	Cardiac magnetic resonance imaging
CS	Coronary sinus
CTO	Chronic total occlusion
CXCL	Chemokine (C-X-C motif) ligand (CXCL5, CXCL6, CXCL8)
ECV	Extracellular volume fraction
EGF	Endothelial growth factor
eGFR	Estimated glomerular filtration rate
EGLN1	Egg-laying defective nine 1
EPO	Erythropoietin
ESR	Erythrocyte sedimentation rate
FDRs	False discovery rates
GDF2	Growth differentiation factor 2
HbA1c	Glycosylated hemoglobin
HBEGF	Heparin-binding EGF-like growth factor
HDL-c	High-density lipoprotein cholesterol
HF	Heart failure
HIF-1α	Hypoxia-inducible factor 1-alpha
hs-CRP	High-sensitivity C-reactive protein
hs-TnI	High-sensitivity troponin I
IL	Interleukin (IL-1β, IL-6, IL-10, IL-27)
IQR	Interquartile range
J-CTO	Japanese chronic total occlusion
LAD	Left anterior descending coronary artery
LCX	Left circumflex coronary artery
LDL-c	Low-density lipoprotein cholesterol
LGE	Late gadolinium enhancement
LVEDV	Left ventricular end diastolic volume
LVEF	Left ventricular ejection fraction
LVESV	Left ventricular end systolic volume
MACEs	Major adverse cardiovascular events
MAPK9	Mitogen-activated protein kinase 9
MI	Myocardial infarction
MLR	Monocyte-to-lymphocyte ratio
MRA	Mineralocorticoid receptor antagonist
NLR	Neutrophil-to-lymphocyte ratio
NSTEMI	Non-ST-segment elevation myocardial infarction
NT-proBNP	N-terminal pro-B-type natriuretic peptide
OLR1	Oxidized low-density lipoprotein receptor 1
PCI	Percutaneous coronary intervention
PCSK9i	Proprotein convertase subtilisin/kexin type 9 inhibitor
PGF	Placenta growth factor
PTX3	Pentraxin 3
P2Y12i	P2Y12 receptor inhibitors
RCA	Right coronary artery
RIPK1	Receptor-interacting protein kinase 1
ROC	Receiver operating characteristic
SD	Standard deviation
SGLT2i	Sodium-glucose cotransporter-2 inhibitor
STEMI	ST-segment elevation myocardial infarction
SYNTAX	Synergy between percutaneous coronary intervention with Taxus and cardiac surgery
THPO	Thrombopoietin
TIMI	Thrombolysis In Myocardial Infarction
TNF	Tumor necrosis factor
UFH	Unfractionated heparin
VEGF	Vascular endothelial growth factor (VEGFA, VEGFD)
VEGFR-2	Vascular endothelial growth factor receptor-2
