# Supplementary material for: Percutaneous Coronary Intervention for Chronic Total Occlusions Modulates Cardiac Hypoxic and Inflammatory Stress
Source: J Clin Med. 2026 Jan 8;15(2):517. doi: 10.3390/jcm15020517 (PMC12841894; doi:10.3390/jcm15020517)
Supplement: Supplementary file 1 [file jcm-15-00517-s001.zip › jcm-4068221-supplementary.pdf]

SUPPLEMENTARY MATERIAL

Olink Flex calibration/normalization, batch/run effects handling, replicate strategy and quality control metrics

Calibration and normalization: Calibration was performed using Olink-provided standard curves and internal controls, ensuring accurate absolute quantification and comparability across plates. Internal assay controls were used to correct for technical variation, and inter-plate normalization was applied according to the manufacture's recommendations.

Batch and run effects: All plates were processed using identical reagents, protocols, and experimental conditions. After normalization, data were inspected to confirm the absence of systematic plate or run effects. No additional batch correction was required, as technical variability between plates was within the validated performance range of the assay.

Replicate strategy: Consistent with Olink Flex validation studies, technical duplicates were not used. Olink Flex assays are designed to operate without duplicates, as extensive validation has demonstrated high analytical precision, with intra- and inter-assay coefficients of variation typically below 10%. The use of internal controls provides robust monitoring of assay performance and reproducibility.

Quality control metrics: Quality control was assessed using Olink's built-in assay and sample controls, including monitoring of assay performance, standard curve behavior, and internal quality control flags. All samples met the predefined quality thresholds, and no samples were excluded due to technical failure. Each sample plate contains eight control samples. Triplicates of the sample control, duplicates of the negative control and triplicates of the calibrator. The calibrator allows for calculation of standard concentration units and is used in a second normalization step. It is designed to improve inter assay precision, enabling optimal comparison of data derived from multiple runs. The sample

control is used to monitor and control the quality of reported output data by evaluating both accuracy and intra assay precision for all assays.

Full details on assay controls, calibration procedures, and quality control metrics are provided in the manufacturer's Olink Flex validation documentation:

https://7074596.fs1.hubspotusercontent-na1.net/hubfs/7074596/01-User%20Manuals%20for%20website/1316-npx-signature-flex-user-manual.pdf

https://7074596.fs1.hubspotusercontent-na1.net/hubfs/7074596/04-Validation%20data/1322-olink-flex-validation-data.pdf

Supplementary Figure S1. ROC curves of coronary sinus biomarkers

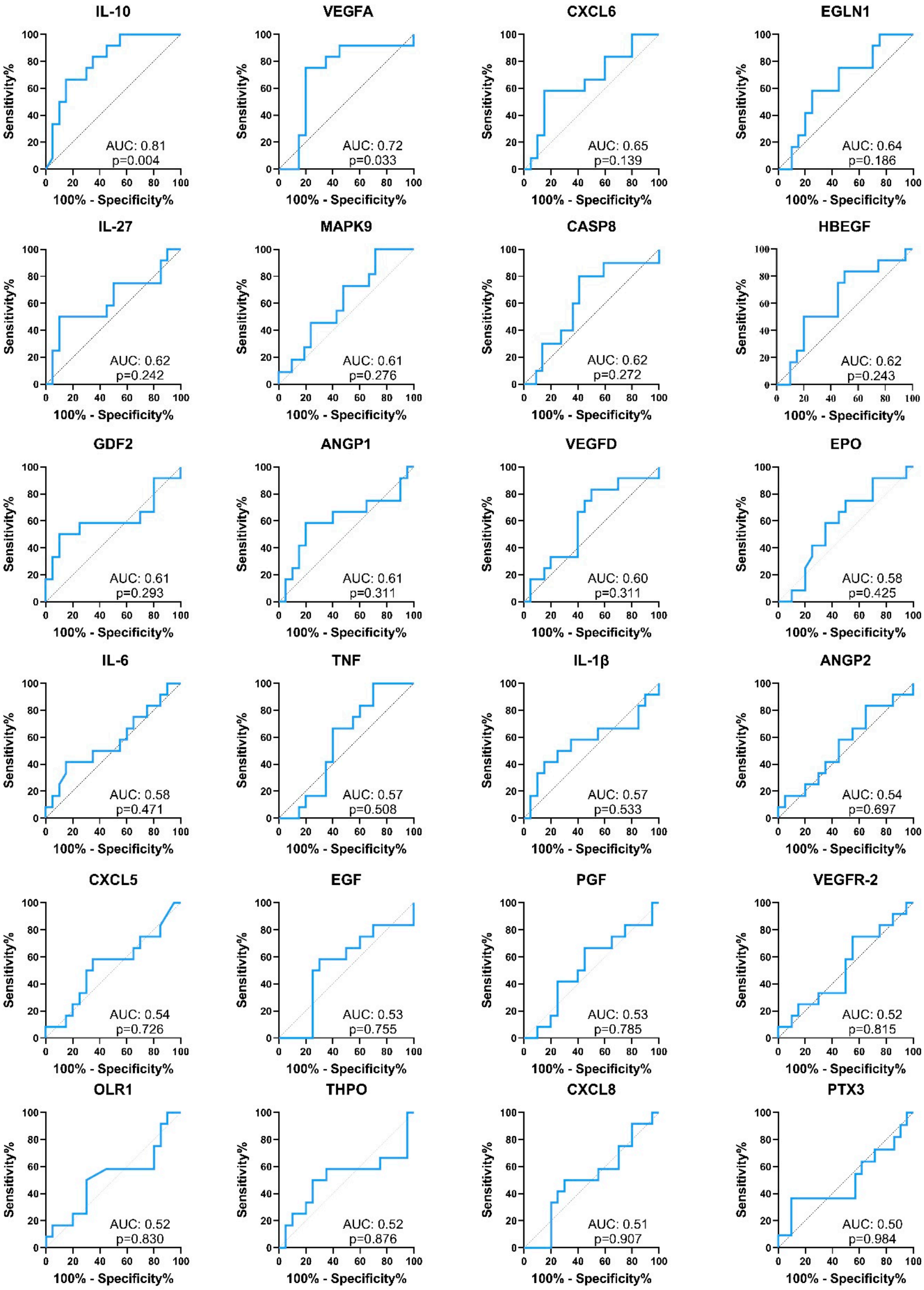

ROC curves depict the discriminative performance of CS biomarkers to predict clinically meaningful improvement of LVEF (≥5 absolute points) after CTO-PCI, based on the results of the 6-month follow-up cardiac magnetic resonance imaging from 33 patients. Sensitivity is plotted on the y-axis and 100-specificity on the x-axis. For each biomarker, the AUC with its raw *P*-value is shown on the plot; higher AUCs reflect better discrimination. Biomarkers are ordered according to their discriminative performance. Detailed data of ROC analysis is provided in *Supplemental Table 6.* ANGP = angiopoietin (ANGP1, ANGP2); AUC = area under the curve; CASP8 = caspase 8; CI = confidence interval; CMR = cardiac magnetic resonance imaging; CS = coronary sinus CTO = chronic total occlusion; CXCL = chemokine (C-X-C motif) ligand (CXCL5, CXCL6, CXCL8); EGF = endothelial growth factor; EGLN1 = egg-laying defective nine 1; EPO = erythropoietin; GDF2 = growth differentiation factor 2; HBEGF = heparin-binding EGF-like growth factor; IL = interleukin (IL-1β, IL-6, IL-10, IL-27); MAPK9 = mitogen-activated protein kinase 9; OLR1 = oxidized low-density lipoprotein receptor 1; PCI = percutaneous coronary intervention; PGF = Placenta growth factor; PTX3 = pentraxin 3; THPO = thrombopoietin; TNF = tumor necrosis factor; VEGF = vascular endothelial growth factor (VEGFA, VEGFD); VEGFR-2 = vascular endothelial growth factor receptor-2

Supplementary Table S1. Descriptive statistics and paired comparisons of coronary sinus biomarkers.

	**Biomarker concentration (pg/ml)**						**Paired comparison**			**Average change (pg/ml)**	
	**Pre CTO-PCI (t_0) (n=33)**	**Post CTO-PCI (t_1) (n=33)**	**LLOQ***	**% below LLOQ**	**ULOQ**	**% above ULOQ**	**Test type**	**Raw *P*-value**	**FDR-adj *P*-value**	**Δ (Post – pre)**	**95 % CI**
	**Hypoxia-associated biomarkers**										
**VEGFD**	1274 ± 464	1094 ± 489	5.7	0	8727	0	Student's *t*	3.2×10^{-8}	8.9×10^{-7}	-180	-230 to -129.4
**VEGFA**	382 (291 - 704)	264.3 (220 - 316)	0.3	0	3130.9	0	Wilcoxon	1.5×10^{-7}	1.6×10^{-6}	-103.9	-192 to -54
**VEGFR-2**	1440 ± 301.6	1253 ± 267.4	1.7	0	16.000	0	Student's *t*	2.5×10^{-7}	1.8×10^{-6}	-187.2	-246 to -128.6
**THPO**	372 (304 - 534.8)	292 (215 - 382)	26.2	0	16.000	0	Wilcoxon	2.2×10^{-5}	1.2×10^{-4}	-63.6	-126.2 to -35
**EPO**	4.5 (2.8 - 10.4)	4.3 (1.8 - 7.1)	0.5	0	735	0	Wilcoxon	6.9×10^{-5}	2.7×10^{-4}	-1.1	-2.1 to -0.35
**EGF**	201.8 (85 - 413.2)	98.6 (53 - 172)	0.3	0	513.2	0	Wilcoxon	2.5×10^{-4}	7.9×10^{-4}	-69.4	-179 to -9.87
**ANGP1**	4658 (2493-10628)	2483 (1323 - 4076)	143.8	0	14046	0	Wilcoxon	0.006	0.015	-1537	-4057 to -183
**EGLN1**	462 (328 - 812)	595 (458 - 1003)	4.9	0	7493	0	Wilcoxon	0.017	0.033	113	15.9 to 447
**GDF2**	51 ± 19	44 ± 17.6	1.7	0	6400	0	Student's *t*	0.031	0.056	-6.9	-13.1 to -0.64

**HBEGF**	28.4 (18.6 - 68)	20.5 (17 - 30.5)	0.2	0	372	0	Wilcoxon	0.032	0.058	-1.2	-15.8 to -1.07
**ANGP2**	3181 ± 775	3326 ± 659	16.7	0	25600	0	Student's *t*	0.060	0.085	144	-55 to 344
**PGF**	88 ± 31	75.4 ± 18	0.1	0	6400	0	Student's *t*	0.073	0.094	-12.5	-26.2 to 12.3
**HIF-1α**†			0.26	91	1024	0					
	**Inflammation-associated biomarkers**										
**IL-10**	4.8 (3.5 - 8)	12.5 (6.9 - 33.3)	0.5	0	4468	0	Wilcoxon	1.8×10^{-7}	1.7×10^{-6}	8.5	2.3 to 15.02
**CXCL8**	257.5 (58 - 1114)	70 (17.2 - 386.7)	0.2	0	1928	1.5	Wilcoxon	6×10^{-5}	2.2×10^{-4}	-190	-413.3 to -52
**OLR1**	1026 (795 - 1302)	723 (451 - 1156)	0.4	0	1423	1.5	Wilcoxon	9.5×10^{-5}	3.3×10^{-4}	-126.6	-341 to -41.8
**CASP8**	72 (45.5 - 103)	50.7 (35 - 64)	0.75	0	2870	0	Wilcoxon	3.2×10^{-8}	0.001	-21	-34 to -10.8
**IL-1β**	2 (0.8 - 12.8)	0.9 (0.5 - 5.8)	0.1	1.5	798	0	Wilcoxon	0.002	0.005	-1.3	-3.26 to -0.63
**IL-27**	14.2 (8.1 - 20.1)	15.6 (9.3 - 26.1)	1.1	0	4275	0	Wilcoxon	0.010	0.022	1.4	0.2 to 4.9
**CXCL6**	117.7 (87 - 151.2)	110.8 (76 - 145)	0.1	0	391	0	Wilcoxon	0.027	0.041	-14	-27.3 to -3.2
**IL-6**	7.5 (3.7 - 22.5)	10.9 (6.1 - 20.5)	0.1	0	1566	0	Wilcoxon	0.134	0.164	3.5	-2.5 to 5.6
**CXCL5**	1572 (847 - 2175)	1349 (711 - 1890)	4	0	2600	1.5	Wilcoxon	0.254	0.284	-40	-240.5 to 12

**PTX3**	1299 (965 - 1610)	1491 (1102 - 1810]	10.5	0	6400	0	Wilcoxon	0.416	0.493	45	-88.9 to 177
**MAPK9**	118.8 (89 - 198.5)	109.3 (73 - 185.7)	22	0	160000	0	Wilcoxon	0.490	0.528	-14.7	-39.7 to 21.5
**TNF**	31 (19.3 - 70)	26.6 (17 - 56)	4.8	0	2922	0	Wilcoxon	0.971	0.984	-3.4	-6.8 to 9.1
**TNFSF12**†			1.75	0	2682	87					

Absolute concentrations (pg/ml) of CS biomarkers from 33 patients are shown before CTO crossing (t_0) and after revascularization (t_1) Values are mean ± SD or median (IQR). Two-tailed *P*-values for paired tests are reported, and FDR is controlled using the Benjamini-Hochberg procedure. Biomarkers are ordered by raw *P*-values. Average change is expressed as mean difference for normally distributed variables or Hodges-Lehmann median difference for non-normally distributed variables, with 95% CI. *Quantification limits of the customizable protein detection system Olink® Flex platform (Olink Proteomics®, Sweden), based on PEA technology, for coronary sinus blood biomarkers. † This biomarker showed most of measurements out of the quantification limits. ANGP = angiopoietin (ANGP1, ANGP2); CASP8 = caspase 8; CI = confidence interval; CS = coronary sinus; CTO = chronic total occlusion; CXCL = chemokine (C-X-C motif) ligand (CXCL5, CXCL6, CXCL8); EGF = endothelial growth factor; EGLN1 = egg-laying defective nine 1; EPO = erythropoietin; FDR = false discovery rate; GDF2 = growth differentiation factor 2; HBEGF = heparin-binding EGF-like growth factor; IL = interleukin (IL-1β, IL-6, IL-10, IL-27); IQR = interquartile range; LLOQ = lower limit of quantitation; MAPK9 = mitogen-activated protein kinase 9; OLR1 = oxidized low-density lipoprotein receptor 1; PEA = proximity extension assay; PCI = percutaneous coronary intervention; PGF = Placenta growth factor;

PTX3 = pentraxin 3; SD = standard deviation; THPO = thrombopoietin; TNF = Tumour necrosis factor; ULOQ = upper limit of quantitation;

VEGF = vascular endothelial growth factor (VEGFA, VEGFD); VEGFR-2 = vascular endothelial growth factor receptor

Supplementary Table S2. Multivariable linear mixed model for changes in coronary sinus biomarkers.

	Estimate (Δ Post – pre) (pg/ml) n=33	95 % CI (pg/ml)	Raw *P*-value	FDR-adj *P*-value
**Hypoxia-associated biomarkers**				
**VEGFD**	-180.1	-228.8 to -131.3	3×10^{-8}	8×10^{-8}
**VEGFR-2**	-187.3	-243.6 to -130.8	3×10^{-7}	3.1×10^{-6}
**EGF**	-109.5	-155.3 to -63.7	5×10^{-5}	2.4×10^{-4}
**VEGFA**	-321.7	-473.7 to -170.2	1.1×10^{-4}	4.5×10^{-4}
**THPO**	-129.7	-192.7 to -66.7	3.3×10^{-4}	5.3×10^{-4}
**EPO**	-1.4	-2.2 to -0.9	7.1×10^{-4}	0.001
**ANGP1**	-2854.5	-4568.4 to -1140.7	0.001	0.003
**HBEGF**	-21.3	-35.2 to -9.4	0.003	0.009
**GDF2**	-6.9	-10.9 to -1.9	0.021	0.035
**EGLN1**	287	110.6 to 493.6	0.031	0.040

**PGF**	-12.5	-23.9 to -0.7	0.042	0.054
**ANGP2**	144.19	-48 to 336	0.110	0.161
**Inflammation-associated biomarkers**				
**IL-10**	13.1	8.4 to 18.1	1.6×10^{-5}	7.8×10^{-5}
**OLR1**	-249.3	-352.6 to -146.1	4×10^{-5}	1.4×10^{-4}
**CASP8**	-23.9	-37.3 to -12.6	4.8×10^{-4}	8.3×10^{-4}
**CXCL8**	-326.3	-495 to -156.9	0.001	0.004
**IL-27**	3	1.7 to 4.3	0.006	0.011
**IL-1β**	-4.2	-6.3 to -2.6	0.009	0.016
**CXCL6**	-13.9	-22.2 to -5.3	0.032	0.044
**CXCL5**	-143.1	-398 to 112	0.280	0.336
**MAPK9**	-5.9	-35 to 23.1	0.690	0.789
**IL-6**	1.8	-9.3 to 13	0.746	0.804
**PTX3**	25	-171.9 to 221.9	0.795	0.855
**TNF**	-3.3	-28.3 to 14	0.804	0.875

Multivariable linear mixed model adjusting the change in the concentration of each CS biomarker for age, sex, baseline biomarker concentration, baseline LVEF, diabetes mellitus, estimated glomerular filtration rate, and key procedural variables (procedural time, CTO location, approach for CTO-crossing, and Rentrop collateral grade), from 33 patients. Estimate represents the mean adjusted change, and 95% CI are provided. Two-tailed *P*-values are reported, and FDR is controlled using the Benjamini-Hochberg procedure. Biomarkers are ordered by raw *P*-values. ANGP = angiopoietin (ANGP1, ANGP2); CASP8 = caspase 8; CI = confidence interval; CS = coronary sinus; CTO = chronic total occlusion; CXCL = chemokine (C-X-C motif) ligand (CXCL5, CXCL6, CXCL8); EGF = endothelial growth factor; EGLN1 = egg-laying defective nine 1; EPO = erythropoietin; FDR = false discovery rate; GDF2 = growth differentiation factor 2; HBEGF = heparin-binding EGF-like growth factor; IL = interleukin (IL-1β, IL-6, IL-10, IL-27); MAPK9 = mitogen-activated protein kinase 9; OLR1 = oxidized low-density lipoprotein receptor 1; PCI = percutaneous coronary intervention; PGF = Placenta growth factor; PTX3 = pentraxin 3; THPO = thrombopoietin; TNF = Tumor necrosis factor; VEGF = vascular endothelial growth factor (VEGFA, VEGFD); VEGFR-2 = vascular endothelial growth factor receptor-2

Supplementary Table S3. Correlation matrix for hypoxia-associated biomarkers.

		ANGP1		ANGP2		VEGFA		VEGFD		VEGFR-2		EGF		HBEGF		GDF2		PGF		THPO		EPO		EGLN1	
		ρ	*P*	ρ	*P*	ρ	*P*	ρ	*P*	ρ	*P*	ρ	*P*	ρ	*P*	ρ	*P*	ρ	*P*	ρ	*P*	ρ	*P*	ρ	*P*
**ANGP1**	**Baseline**			0.08	.763	0.77	1.5X 10^{-4}	0.09	.823	0.27	.327	0.91	3.4X 10^{-5}	0.93	6.4X 10^{-6}	0.45	.119	(-) 0.43	.111	0.58	.044	0.17	.457	0.10	.617
	**Δ Post-pre**			0.38	.290	0.68	.002	0.47	.160	0.46	.180	0.79	3.6X 10^{-4}	0.94	7.9X 10^{-7}	0.55	.043	(-) 0.55	.041	0.71	4.3X 10^{-4}	0.15	.420	(-) 0.45	.112
**ANGP2**	**Baseline**	0.08	.763			0.16	.496	0.43	.138	0.22	.444	0.10	.596	0.11	.563	(-) 0.01	.925	0.18	.520	(-) 0.06	.783	(-) 0.12	.586	0.04	.845
	**Δ Post-pre**	0.38	.290			0.53	.054	0.49	.168	0.44	.153	0.35	.290	0.44	.190	0.51	.059	0.09	.705	0.56	.049	0.33	.223	(-) 0.14	.563
**VEGFA**	**Baseline**	0.77	1.5X 10^{-4}	0.16	.496			0.04	.840	0.51	.049	0.74	8.7X 10^{-4}	0.80	1.8X 10^{-4}	0.53	.039	(-) 0.51	.058	0.51	.061	0.17	.436	0.04	.793
	**Δ Post-pre**	0.68	.002	0.53	.054			0.42	.160	0.78	6.3X 10^{-4}	0.58	.032	0.72	.001	0.81	1.9X 10^{-4}	(-) 0.43	.130	0.84	1.2X 10^{-4}	0.58	.035	(-) 0.30	.330
**VEGFD**	**Baseline**	0.09	.823	0.43	.138	0.04	.840			0.01	.940	(-) 0.02	.890	0.01	.957	0.07	.795	(-) 0.04	.832	(-) 0.11	.536	0.05	.763	(-) 0.04	.842
	**Δ Post-pre**	0.47	.160	0.49	.168	0.42	.160			0.55	.034	0.47	.178	0.45	.163	0.46	.158	(-) 0.04	.804	0.48	.183	0.35	.223	(-) 0.37	.190
**VEGFR-2**	**Baseline**	0.27	.327	0.22	.444	0.51	.049	0.01	.940			0.34	.392	0.33	.353	0.45	.192	(-) 0.29	.414	0.22	.450	(-) 0.02	.900	0.08	.821
	**Δ Post-pre**	0.46	.180	0.44	.153	0.78	6.3X 10^{-4}	0.55	.034			0.43	.130	0.49	.106	0.79	3.9X 10^{-4}	(-) 0.26	.339	0.66	.008	0.61	.016	(-) 0.20	.343
**EGF**	**Baseline**	0.91	3.4X 10^{-5}	0.10	.596	0.74	8.7X 10^{-4}	(-) 0.02	.890	0.34	.192			0.88	1.8X 10^{-5}	0.31	.290	(-) 0.41	.133	0.51	.056	0.21	.333	0.25	.460
	**Δ Post-pre**	0.79	3.6X 10^{-4}	0.35	.290	0.58	.032	0.47	.178	0.43	.130			0.72	8.8X 10^{-4}	0.49	.153	(-) 0.44	.102	0.73	6.3X 10^{-4}	0.17	.452	(-) 0.40	.200
**HBEGF**	**Baseline**	0.93	6.4X 10^{-6}	0.11	.563	0.80	1.8X 10^{-4}	0.01	.957	0.33	.353	0.88	1.8X 10^{-5}			0.41	.128	(-) 0.33	.293	0.58	.043	0.21	.380	0.11	.590
	**Δ Post-pre**	0.94	7.9X 10^{-7}	0.44	.190	0.72	.001	0.45	.163	0.49	.106	0.72	8.8X 10^{-4}			0.66	.003	(-) 0.42	.156	0.77	2.2X 10^{-4}	0.17	.422	(-) 0.41	.180

**GDF2**	**Baseline**	0.45	.119	- 0.01	.925	0.53	.039	0.07	.795	0.45	.192	0.31	.290	0.41	.128			(-) 0.40	.190	0.48	.098	0.07	.703	(-) 0.22	.332
	**Δ Post-pre**	0.55	.043	0.51	.059	0.81	1.9X10^{-4}	0.46	.158	0.79	3.9X10^{-4}	0.49	.153	0.66	.003			(-) 0.23	.364	0.82	9.2X10^{-5}	0.51	.053	(-) 0.32	.262
**PGF**	**Baseline**	(-) 0.43	.111	0.18	.520	(-) 0.51	.058	(-) 0.04	.832	(-) 0.29	.414	(-) 0.41	.133	(-) 0.33	.293	(-) 0.40	.190			(-) 0.56	.032	0.11	.560	(-) 0.08	.868
	**Δ Post-pre**	(-) 0.55	.041	0.09	.705	(-) 0.43	.130	(-) 0.04	.804	(-) 0.26	.339	(-) 0.44	.102	(-) 0.42	.156	(-) 0.23	.364			(-) 0.44	.100	(-) 0.05	.756	(-) 0.01	.954
**THPO**	**Baseline**	0.58	.044	(-) 0.06	.783	0.51	.061	(-) 0.11	.536	0.22	.450	0.51	.056	0.58	.043	0.48	.098	(-) 0.56	.032			0.05	.786	0.01	.987
	**Δ Post-pre**	0.71	4.3X10^{-4}	0.56	.049	0.84	1.2X10^{-4}	0.48	.183	0.66	.008	0.73	6.3X10^{-4}	0.77	2.2X10^{-4}	0.82	9.2X10^{-5}	(-) 0.44	.100			0.41	.116	(-) 0.24	.377
**EPO**	**Baseline**	0.17	.457	(-) 0.12	.586	0.17	.436	0.05	.763	(-) 0.02	.900	0.21	.333	0.21	.380	0.07	.703	0.11	.560	0.05	.786			(-) 0.23	.392
	**Δ Post-pre**	0.15	.420	0.33	.223	0.58	.035	0.35	.223	0.61	.016	0.17	.452	0.17	.422	0.51	.053	(-) 0.05	.756	0.41	.116			(-) 0.24	.372
**EGLN1**	**Baseline**	0.10	.617	0.04	.845	0.04	.793	(-) 0.04	.842	0.08	.821	0.25	.460	0.11	.590	(-) 0.22	.332	(-) 0.08	.868	0.01	.987	(-) 0.23	.392		
	**Δ Post-pre**	(-) 0.45	.112	(-) 0.14	.563	(-) 0.30	.330	(-) 0.37	.190	(-) 0.20	.343	(-) 0.40	.200	(-) 0.41	.180	(-) 0.32	.262	(-) 0.01	.954	(-) 0.24	.377	(-) 0.24	.372		

Correlation matrix corresponding to *Figure 3*. Spearman's coefficients (ρ) and FDR-adjusted *P*-values are shown for correlations among baseline levels (before CTO-PCI) and changes (delta post-pre revascularization) in CS blood concentration of hypoxia-associated biomarkers from 33 patients. Strongest correlations (│ρ│ ≥ 0.65 and FDR-adjusted *P* < 0.01) are highlighted in red for clarity. ANGP = angiopoietin (ANGP1, ANGP2); CS = coronary sinus; CTO = chronic total occlusion; EGF = endothelial growth factor; EGLN1 = egg-laying defective nine 1; EPO = erythropoietin; FDR = false discovery rate; GDF2 = growth differentiation factor 2; HBEGF = heparin-binding EGF-like growth factor; PCI = percutaneous coronary intervention; PGF = Placenta growth factor; THPO = thrombopoietin; VEGF = vascular endothelial growth factor (VEGFA, VEGFD); VEGFR-2 = vascular endothelial growth factor receptor-2

Supplementary Table S4. Correlation matrix for inflammation-associated biomarkers.

		IL-10		IL-27		IL-1β		IL-6		TNF		CXCL5		CXCL6		CXCL8		CASP8		MAPK9		PTX3		OLR1	
		ρ	*P*	ρ	*P*	ρ	*P*	ρ	*P*	ρ	*P*	ρ	*P*	ρ	*P*	ρ	*P*	ρ	*P*	ρ	*P*	ρ	*P*	ρ	*P*
**IL-10**	**Baseline**			0.53	.048	0.03	.861	0.17	.441	0.13	.562	0.04	.805	0.39	.230	0.03	.841	0.10	.558	0.33	.270	0.14	.431	0.03	.863
	**Δ Post-pre**			0.58	.039	0.05	.773	0.46	.134	0.22	.314	(-) 0.10	.576	0.01	.918	0.03	.301	0.18	.517	0.26	.187	0.26	.637	0.35	.305
**IL-27**	**Baseline**	0.53	.048			0.10	.581	0.07	.691	0.08	.635	0.15	.394	0.56	.047	0.04	.842	0.23	.393	0.26	.238	0.25	.351	0.01	.960
	**Δ Post-pre**	0.58	.039			0.02	.916	0.47	.102	0.25	.366	0.05	.799	0.28	.310	0.03	.854	0.12	.517	0.23	.287	(-) 0.08	.637	0.23	.305
**IL-1β**	**Baseline**	0.03	.861	0.03	.863			0.75	3.8X 10^{-4}	0.72	4.3X 10^{-4}	0.21	.384	0.24	.300	0.90	7.4X 10^{-6}	0.28	.308	0.55	.035	0.67	9.7X 10^{-4}	0.51	.037
	**Δ Post-pre**	0.05	.773	0.35	.305			0.33	.138	0.12	.489	0.52	.051	0.43	.112	0.77	2.3X 10^{-4}	0.47	.116	0.56	.031	0.41	.117	(-) 0.17	.531
**IL-6**	**Baseline**	0.17	.441	0.07	.691	0.75	3.8X 10^{-4}			0.59	.041	0.19	.320	0.16	.430	0.79	4.9X 10^{-4}	0.07	.703	0.24	.282	0.35	.263	0.40	.124
	**Δ Post-pre**	0.46	.134	0.47	.102	0.33	.138			0.30	.280	0.23	.306	0.32	.295	0.51	.053	0.27	.286	0.46	.116	0.25	.353	0.18	.415
**TNF**	**Baseline**	0.13	.562	0.08	.635	0.72	4.3X 10^{-4}	0.59	.041			0.26	.185	0.24	.210	0.70	9.2X 10^{-4}	0.1	.590	0.35	.062	0.56	.005	0.50	.008
	**Δ Post-pre**	0.22	.314	0.25	.366	0.12	.489	0.30	.280			0.03	.849	0.33	.230	0.30	.280	0.20	.370	0.26	.349	0.14	.440	0.29	.284
**CXCL5**	**Baseline**	0.04	.805	0.15	.394	0.21	.384	0.19	.320	0.26	.185			0.25	.170	0.20	.290	0.40	.143	0.36	.262	0.35	.260	0.38	.252
	**Δ Post-pre**	(-) 0.10	.576	0.05	.799	0.52	.051	0.23	.306	0.03	.849			0.66	.009	0.42	.113	0.30	.287	0.49	.102	0.10	.588	(-) 0.23	.391
**CXCL6**	**Baseline**	0.39	.230	0.56	.047	0.24	.300	0.16	.430	0.24	.210	0.25	.170			0.22	.336	0.26	.343	0.26	.358	0.37	.272	0.05	.800
	**Δ Post-pre**	0.01	.918	0.28	.310	0.43	.112	0.32	.295	0.33	.230	0.66	.009			0.57	.049	0.44	.110	0.53	.052	0.13	.580	(-) 0.03	.869

**CXCL8**	**Baseline**	0.03	.841	0.04	.842	0.90	7.4X 10^{-6}	0.79	4.9X 10^{-4}	0.70	9.2X 10^{-4}	0.20	.290	0.22	.336			0.27	.389	0.42	.116	0.53	.039	0.58	.032
	**Δ Post-pre**	0.03	.301	0.03	.854	0.77	2.3X 10^{-4}	0.51	.053	0.30	.280	0.42	.113	0.57	.049			0.43	.112	0.55	.042	0.41	.117	(-) 0.16	.562
**CASP8**	**Baseline**	0.10	.558	0.23	.393	0.28	.308	0.07	.703	0.1	.590	0.40	.143	0.26	.343	0.27	.389			0.75	5.2X 10^{-4}	0.34	.220	0.40	.110
	**Δ Post-pre**	0.18	.517	0.12	.517	0.47	.116	0.27	.286	0.20	.370	0.30	.287	0.44	.110	0.43	.112			0.78	4.4X 10^{-4}	0.60	.011	0.37	.238
**MAPK 9**	**Baseline**	0.33	.270	0.26	.238	0.55	.035	0.24	.282	0.35	.062	0.36	.262	0.26	.358	0.42	.116	0.75	5.2X 10^{-4}			0.52	.043	0.36	.256
	**Δ Post-pre**	0.26	.187	0.23	.287	0.56	.031	0.46	.116	0.26	.349	0.49	.102	0.53	.052	0.55	.042	0.78	4.4X 10^{-4}			0.65	.009	0.30	.281
**PTX3**	**Baseline**	0.14	.431	0.25	.351	0.67	9.7X 10^{-4}	0.35	.263	0.56	.005	0.35	.260	0.37	.272	0.53	.039	0.34	.220	0.52	.043			0.65	012
	**Δ Post-pre**	0.26	.637	(-) 0.08	.637	0.41	.117	0.25	.353	0.14	.440	0.10	.588	0.13	.580	0.41	.117	0.60	.011	0.65	.009			.024	.383
**OLR1**	**Baseline**	0.03	.863	0.35	.305	0.51	.037	0.40	.124	0.50	.008	0.38	.252	0.05	.800	0.58	.032	0.40	.110	0.36	.256	0.65	.012		
	**Δ Post-pre**	0.35	.305	0.01	.960	(-) 0.17	.531	0.18	.415	0.29	.284	(-) 0.23	.391	(-) 0.03	.869	(-) 0.16	.562	0.37	.238	0.30	.281	0.24	.383		

Correlation matrix corresponding to *Figure 4*. Spearman's coefficients (ρ) and FDR-adjusted *P*-values are shown for correlations among baseline levels (before CTO-PCI) and changes (delta post-pre revascularization) in CS blood concentration of inflammation-associated biomarkers. Pre and post samples were taken from 33 patients. Strongest correlations ($|\rho| \geq 0.65$ and FDR-adjusted $P < 0.01$) are highlighted in red for clarity. CASP8 = caspase 8; CS = coronary sinus; CTO = chronic total occlusion; CXCL: chemokine (C-X-C motif) ligand (CXCL5, CXCL6, CXCL8); FDR = false discovery rate; IL = interleukin (IL-1β, IL-6, IL-10, IL-27); MAPK9 = mitogen-activated protein kinase 9; OLR1 = oxidized low-density lipoprotein receptor 1; PCI = percutaneous coronary intervention; PTX3 = pentraxin 3; TNF = Tumor necrosis factor

Supplementary Table S5. Changes in systemic biomarkers and CMR parameters 6 months after CTO-PCI

	Pre CTO-PCI (n=33)	Post CTO-PCI (n=33)	Paired test			Average change	
			**Test type**	**Raw *P*-value**	**FDR-adj *P*-value**	**Δ (Post – pre)**	**95 % CI**
**Laboratory parameters**							
**NT-proBNP (pg/ml)**	560 (241 - 1027)	318 (137 – 572)	Wilcoxon	1.3×10^{-4}	0.007	-206	-376 to -52
**hsTnI (ng/L)**	9.4 ± 4.94	5.93 ± 3.91	Student's *t*	8.9×10^{-4}	0.010	-3.2	-5.24 to -1.16
**hsCRP (mg/L)**	3.5 (1.5 - 4.9)	1 (0.3 – 2.5)	Wilcoxon	0.001	0.016	-1.93	-3.43 to -0.55
**NLR**	2.7 (2 – 4.2)	2.4 (1.6 – 3.3)	Wilcoxon	0.004	0.028	-0.26	-0.41 to -0.11
**ESR (mm/h)**	9 (6 – 16)	5 (3 – 10.50)	Wilcoxon	0.005	0.036	-2	- 4 to -1
**MLR**	0.34 (0.26 – 0.45)	0.30 (0.23 – 0.36)	Wilcoxon	0.064	0.112	-0.08	-0.10 to 0.02
**CMR parameters**							
**Native T1 (ms)**	1050 ± 84.77	1010 ± 62.28	Student's *t*	0.002	0.016	-40	-64.3 to -15.67

**LVEF (%)**	51.15 ± 10	53.58 ± 10.2	Student’s *t*	0.007	0.028	2.4	0.70 to 4.1
**Native T2 (ms)**	45 (43.5 – 54.5)	45 (41.5 – 50.5)	Wilcoxon	0.029	0.078	-1	-3 to 0
**ECV (%)**	27 (25.5 to 32.5)	26 (23.5 to 28.5)	Wilcoxon	0.040	0.112	-1	-3 to 1
**LVESV (ml)**	80 (63.5 – 102)	69 (58 – 107.5)	Wilcoxon	0.218	0.292	-2	-11 to 3
**LVEDV (ml)**	173.8 ± 41.23	173.7 ± 44	Student’s *t*	0.901	0.970	-0.12	-9.4 to 9.2

Changes in laboratory and CMR parameters from 33 patients are shown before CTO-PCI and six months after the revascularization procedure. Values are mean ± SD or median (IQR). Two-tailed *P*-values for paired tests are reported. Biomarkers are ordered by raw *P*-values. Average change is expressed as mean difference for normally distributed variables or Hodges-Lehmann median difference for non-normally distributed variables, with 95% CI. CI = confidence interval; CMR = cardiac magnetic resonance imaging; CTO = chronic total occlusion; ECV = extracellular volume; ESR = erythrocyte sedimentation rate; hsCRP = high-sensitivity C-reactive protein; hsTnI = high-sensitivity troponin I; LVEDV = left ventricular end diastolic volume; LVEF = left ventricular ejection fraction; LVESV = left ventricular end systolic volume; MLR = monocyte-to-lymphocyte ratio; NLR = neutrophil to lymphocyte ratio; NT-proBNP = N-terminal pro-B-type natriuretic peptide; PCI = percutaneous coronary intervention

Supplementary Table S6. ROC analysis of coronary sinus biomarkers for predicting left ventricular ejection fraction improvement

	AUC	95% CI	*P*-value	Best cut-off point					
				Value (pg/ml)	Sensitivity (%)	95% CI	Specificity (%)	95% CI	LR
**IL-10**	0.81	0.66 to 0.96	0.004	>5.45	67	40 to 86	85	64 to 95	4.44
**VEGFA**	0.72	0.52 to 0.92	0.033	>406.32	73	47 to 91	80	58 to 91	3.75
**CXCL6**	0.65	0.46 to 0.86	0.139	>139	58	32 to 81	85	64 to 95	3.89
**EGLN1**	0.64	0.44 to 0.84	0.186	<437	58	31 to 81	75	53 to 88	2.33
**IL-27**	0.62	0.41 to 0.84	0.242	>18.54	50	25 to 74	90	70 to 98	5
**HBEGF**	0.62	0.40 to 0.84	0.243	> 20.34	83	55 to 97	50	30 to 70	1.67
**CASP8**	0.62	0.41 to 0.92	0.272	>67.14	80	49 to 96	59	39 to 77	1.87
**MAPK9**	0.61	0.41 to 0.85	0.276	> 109.9	72	43 to 90	52	32 to 71	1.52
**GDF2**	0.61	0.38 to 0.84	0.293	>54.70	58	32 to 80	75	53 to 88	2.33
**ANGP1**	0.61	0.39 to 0.83	0.311	>6936	58	32 to 82	75	58 to 92	2.90
**VEGFD**	0.60	0.41 to 0.91	0.335	>1055	53	55 to 97	50	30 to 70	1.67
**EPO**	0.58	0.38 to 0.79	0.425	>3.40	75	47 to 91	50	29 to 70	1.50
**IL-6**	0.58	0.36 to 0.79	0.471	<3.7	42	19 to 68	85	64 to 94	2.78
**TNF**	0.57	0.37 to 0.77	0.508	<69.50	83	55 to 97	40	22 to 61	1.38
**IL-1β**	0.57	0.34 to 0.79	0.533	<1.04	50	25 to 76	75	53 to 89	2
**ANGP2**	0.54	0.33 to 0.75	0.697	>2830	85	55 to 97	35	18 to 57	1.28
**CXCL5**	0.54	0.32 to 0.75	0.726	<1334	58	31 to 80	65	43 to 82	1.67

**EGF**	0.53	0.31 to 0.75	0.755	>281.80	58	32 to 80	70	48 to 85	1.94
**PGF**	0.53	0.32 to 0.74	0.785	>78.96	67	39 to 86	50	30 to 70	1.33
**VEGFR-2**	0.52	0.31 to 0.74	0.815	>1357	75	47 to 91	45	26 to 66	1.36
**OLR1**	0.52	0.30 to 0.74	0.830	>953	50	25 to 75	70	48 to 75	1.67
**THPO**	0.52	0.28 to 0.75	0.876	<321	50	25 to 75	75	53 to 89	2
**CXCL8**	0.51	0.30 to 0.72	0.901	<124.5	50	25 to 75	70	48 to 85	1.67
**PTX3**	0.50	0.27 to 0.73	0.984	>983	73	43 to 90	28	14 to 50	1.10

ROC analysis exploring the discriminative performance of coronary sinus biomarkers to predict clinically meaningful improvement of LVEF (≥5 absolute points) after percutaneous coronary intervention of chronic total occlusion, based on the results of the 6-month follow-up CMR from 33 patients. For each biomarker, the area under curve (AUC) with 95% confidence interval (CI) and corresponding *P*-value (calculated using the DeLong's method) are reported. The optimal cut-off point (determined by the Youden index), sensitivity and specificity with 95% CI (calculated with the Wilson-Brown method), and likelihood ratio, are reported. Biomarkers are ranked according to AUC and *P*-value. ANGP = angiopoietin (ANGP1, ANGP2); AUC = area under curve; CASP8 = caspase 8; CI = confidence interval; CMR = cardiac magnetic resonance imaging; CXCL: chemokine (C-X-C motif) ligand (CXCL5, CXCL6, CXCL8); EGF = endothelial growth factor; EPO = erythropoietin; GDF2 = growth differentiation factor 2; HBEGF = heparin-binding EGF-like growth factor; IL = interleukin (IL-1β, IL-6, IL-10, IL-27); LR: likelihood ratio; MAPK9 = mitogen-activated protein kinase 9; OLR1 = oxidized low-density lipoprotein receptor 1; PGF = Placenta growth factor; PTX3 = pentraxin 3; THPO = thrombopoietin; ROC: receiver operating characteristic; TNF = Tumor necrosis factor; VEGF = vascular endothelial growth factor (VEGFA, VEGFD); VEGFR-2 = vascular endothelial growth factor receptor

Supplementary Table S7. Discriminative performance across different interleukin-10 cutoff points for predicting left ventricular ejection fraction improvement

Cut-off point for IL-10 (pg/mL)	Sensitivity (%)	95% CI	Specificity (%)	95% CI	LR
> 1.93	100	76 to 100	5	1 to 24	1.05
> 2.48	100	76 to 100	10	2 to 30	1.11
> 2.90	100	76 to 100	15	5 to 36	1.17
> 3.21	100	76 to 100	25	11 to 47	1.33
> 3.30	100	76 to 100	30	14 to 52	1.42
> 3.35	100	76 to 100	35	18 to 57	1.53
> 3.42	100	76 to 100	40	22 to 61	1.67
> 3.52	100	76 to 100	45	26 to 66	1.82
> 3.57	92	65 to 99	45	26 to 66	1.67
> 3.66	92	65 to 99	50	30 to 70	1.83
> 3.84	92	65 to 99	55	34 to 74	2
> 4.02	83	55 to 97	55	34 to 74	1.85
> 4.32	83	55 to 97	60	39 to 78	2
> 4.66	83	55 to 97	65	43 to 82	2.41
> 4.80	75	47 to 91	65	43 to 82	2.14
> 4.85	75	47 to 91	70	48 to 85	2.50
> 4.94	67	39 to 86	70	48 to 85	2.22

> 5	67	39 to 86	75	53 to 89	2.67
> 5.14	67	39 to 86	80	58 to 91	3,333
> 5.45	67	39 to 86	85	64 to 95	4.44
> 5.69	58	32 to 81	85	64 to 95	3.89
> 6.80	50	25 to 75	85	64 to 95	3.33
> 7.90	50	25 to 75	90	69 to 98	5
> 8.00	41	19 to 68	90	69 to 98	4.2
> 8.14	33	14 to 61	90	69 to 98	3.33
> 9.06	33	14 to 61	95	76 to 100	6.67
> 10.94	25	9 to 53	95	76 to 100	5
> 12.17	17	3 to 45	95	76 to 100	3.33
> 12.85	8	1 to 35	95	76 to 100	1.67

Discriminative performance of baseline interleukin-10 (IL-10) levels for predicting left ventricular ejection fraction (LVEF) improvement, across different cut-off points. IL-10 baseline levels were measured in coronary sinus blood samples. Clinically relevant improvement of LVEF was defined as an increase of ≥5 absolute points on cardiac magnetic resonance imaging at 6-month follow-up. For each cut-off point, the sensitivity and specificity values are provided with 95% confidence interval (calculated with the Wilson-Brown method), together with the corresponding likelihood ratio. CI = confidence interval; IL-10 = interleukin-10; LR = likelihood ratio; LVEF = left ventricular ejection fraction
